# Deciphering the mechanism of γ-cyclodextrin’s hydrophobic cavity hydration: an integrated experimental and theoretical study

**DOI:** 10.3762/bjoc.20.221

**Published:** 2024-10-17

**Authors:** Stiliyana Pereva, Stefan Dobrev, Tsveta Sarafska, Valya Nikolova, Silvia Angelova, Tony Spassov, Todor Dudev

**Affiliations:** 1 Faculty of Chemistry and Pharmacy, Sofia University “St. Kliment Ohridski”, 1164 Sofia, Bulgaria; 2 Institute of Optical Materials and Technologies “Acad. J. Malinowski”, Bulgarian Academy of Sciences, 1113 Sofia, Bulgaria; 3 University of Chemical Technology and Metallurgy, 8 St. Kliment Ohridski Blvd, 1756 Sofia, Bulgaria

**Keywords:** cyclodextrin, DFT calculation, DSC/TG, hydration, thermodynamic characteristic

## Abstract

Cyclodextrins (CDs) are host systems with inherent capability for inclusion complex formation with various molecular entities, mostly hydrophobic substances. Host CDs are highly accommodative to water molecules as well and usually contain water in the native state. There is still an ongoing discussion on both the total number of water molecules and their preferred binding position inside the cavities of the CDs. To understand the hydration/dehydration properties of γ-CD (the largest of the three most abundant native CDs), the main experimental methods applied in this study were differential scanning calorimetry (DSC) and thermogravimetric analysis (TGA). By coupling these techniques with density functional theory (DFT) calculations we try to shed some light on the mechanism of the γ-CD hydration and to address some unanswered questions: (i) what are the preferable locations for water molecules in the macrocyclic cavity (“hot spots”); (ii) what are the major factors contributing to the stability of the water cluster in the CD interior; (iii) what type of interactions (i.e., water–water and/or water–CD walls) contribute to the stability of the water assemble; (iv) how does the mechanism of the γ-CD hydration compare with those of its α-CD and β-CD counterparts.

## Introduction

Cyclodextrins (CDs), the remarkable macrocyclic molecules with significant impact on our daily life, have completed their 130th anniversary in 2021 [1]. These cavitands are made of 6–9 glucose fragments, linked with 1-4 α-glycosidic bonds. Most common is the β-CD with 7 glucose residues, but α-CDs with 6 and γ-CDs with 8 glucopyranose units, respectively, have also been widely used as potent host structures encapsulating various substances of interest to science and industry. The shape of CDs is toroid-like with one opening (lower rim) wider than the other (upper rim). These sides, unlike the internal cavity, are hydrophilic, decorated with primary (narrow rim) and secondary (wider rim) hydroxy groups. Van der Waals (vdW) and hydrophobic interactions have been identified as the main driving forces for CDs inclusion complex formation [2–4]. Electrostatic interactions and hydrogen bonding, although generally not dominant, can influence the complex formation as well [5]. Cyclodextrins form inclusion complexes with polar and non-polar substances of various aggregate states. This incredible versatility, combined with the enhanced stability against oxidation, as well as increased solubility of the entrapped molecules, led to the wide range of applications in the pharmaceutical, food, cosmetic, agricultural, and other industries.

It is well established that in the absence of other candidates, water molecules fill the CDs void [6–10]. The number and geometric placement/locality of these water molecules in α-, β-, and γ-CDs is a matter of continuing debate. The water ligands could be coordinated inside the cavity or around either rim. Note that CDs retain some water molecules (numbers and position depending on the nature of the guest molecule) after the inclusion complex is formed [11]. This suggests that hydration of CDs remains an important factor, especially given a recent study that showed how hydration history contributes to the hydration ability of α-CD [12]. Numerous experimental and computational studies have investigated the hydration of natural and substituted/modified CDs. Recent studies combining experimental methods with molecular modeling have revealed that the maximum number of water molecules entrapped inside the macrocyclic cavity is 6 for α-CD [13] and 10 for β-CD [14]. Notably, the exact number of the encapsulated water molecules by γ-CD and the mechanism of its hydration is still a matter of controversial discussion. Too much of a surprise for the scientific community, it has been reported that γ-CD, having the largest cavity (≈9–10 Å in inner diameter) compared to α-CD and β-CD (≈5–6 and ≈7–8 Å in inner diameter, respectively [1,15–16]), may accommodate internally fewer water molecules than β-CD. The number of experimentally identified water molecules for γ-CD varies widely (between 5 and 17) [17], as the hydrating water molecules have high mobility inside the γ-CD cavity [18–19]. This discrepancy highlights the need for additional studies to elucidate the mechanism of the γ-CD hydration. Herewith, by employing a combination of experimental (differential scanning calorimetry/thermogravimetry) and theoretical approaches (density functional theory calculations) we endeavor to shed additional light on the mechanism of the γ-CD hydration. Some unanswered questions have been addressed, more specifically (i) what are the preferable locations for water molecules in the macrocyclic cavity (“hot spots”); (ii) what are the major factors contributing to the stability of the water cluster in the CD interior; (iii) what type of interactions (i.e. water–water and/or water–CD walls) contribute to the stability of the water assemble; (iv) how does the mechanism of the γ-CD hydration compare with those of its α-CD and β-CD counterparts. Our findings illuminate the mechanism of γ-CD hydration and disclose the main factors controlling the process. An occupancy of up to 7 hydration water molecules has been found and a comparison between α-CD, β-CD, and γ-CD hydration mechanisms is provided.

## Results and Discussion

### Nonhydrated γ-CD

**Figure 1:**
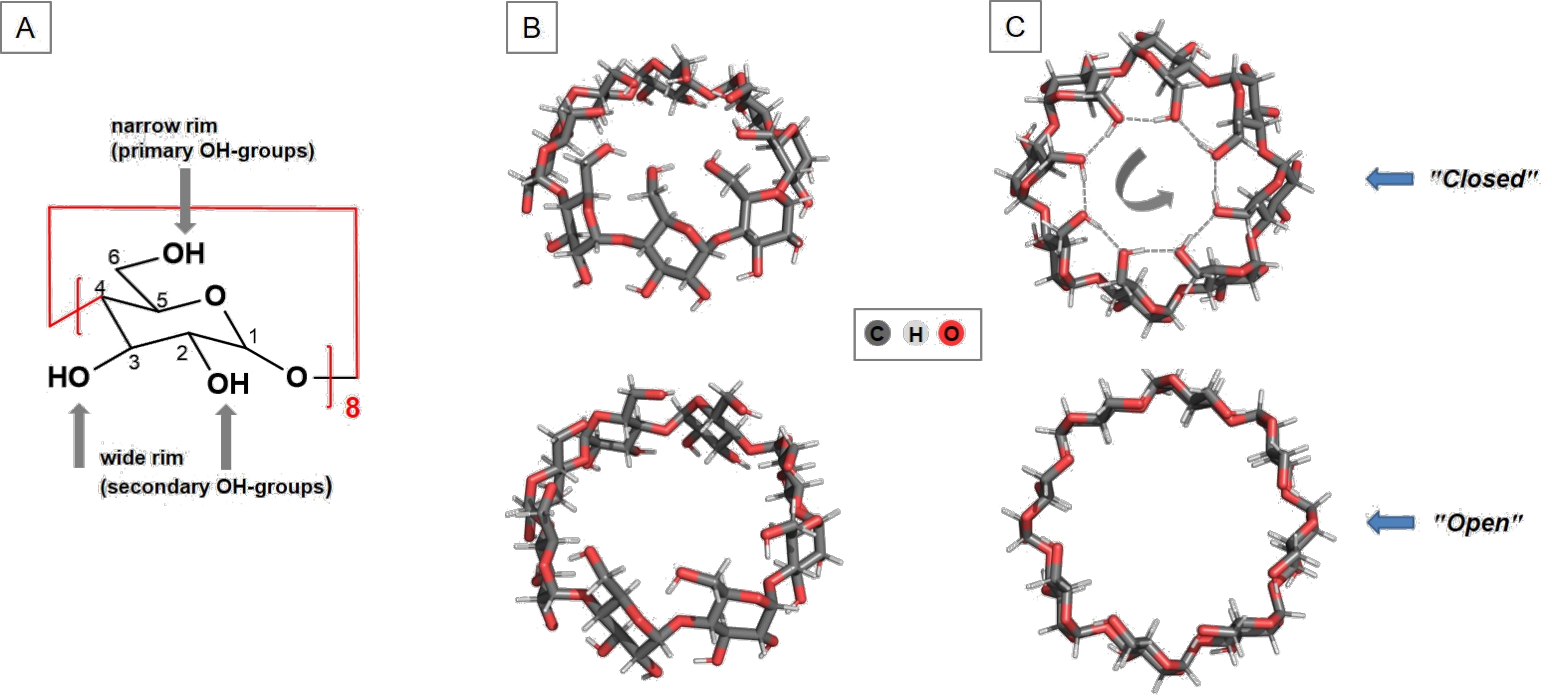
Chemical structure of γ-CD (A); M062X/6-31G(d,p) optimized conformers of nonhydrated γ-CD in two projections – side view (B) and top view from O6 side of the truncated cone (C). The "closed" conformation is with oppositely oriented intramolecular hydrogen bonds on the two edges: viewed from above (the narrow rim side; O6 side), the orientation of the hydrogen bonds on the wide rim (O2/O3 side of the truncated cone) is clockwise (CW), while the orientation of the hydrogen bonds on the narrow rim is counterclockwise (CCW).

As with the other two cyclodextrins (α- and β-), before proceeding to the complexes with water, we considered two main possible conformers of the unhydrated γ-CD with differently oriented primary hydroxy groups. As stated earlier, all native CDs are lined with primary and secondary hydroxy groups (Figure 1). The presence of multiple closely spaced OH groups enables these groups to participate in intramolecular hydrogen bonds between themselves, with water molecules in aqueous solution, or with guest molecules that fit into host’s cavity [20].

The primary hydroxy groups in the nonhydrated γ-CD (located in the upper/narrow rim; O6 side of the truncated cone) can be coordinated (arranged) in two ways (Figure 1): (1) facing inward into the cavity, which creates a belt of hydrogen bonds (the so-called “head–tail” arrangement, “closed” conformation). Notably, in this way, the size of the cavity vestibule is significantly reduced and in the second way (2) they are directed outside the cavity, thus enlarging the aperture (“open” configuration). In the “open” configuration, the primary hydroxy groups are not involved in the intramolecular hydrogen-bonding interactions with neighboring OH groups. Relatively weak hydrogen bonds are formed between the secondary hydroxy groups at the wider/lower rim of the molecule (O2/O3 side of the truncated cone). These hydrogen bonds have been detected experimentally by NMR: it was supposed that the OH-3 group of one glucose monomer is interacting with the OH-2 group of the neighboring glucose unit [21–22]. Experimental evidence for the involvement of primary hydroxy groups in intramolecular H-bonds is contradictory and confusing – observations suggest no involvement of secondary hydroxy protons in intramolecular H-bonds and strong exposure of OH-6 groups to the solvent [22].

Our calculations reveal (as expected) that the “closed” narrow-rim arrangement is energetically more favorable than the “open” configuration (by 22.3 kcal mol^−1^), which is why the “head–tail” structure was used in subsequent evaluations. It should be noted that for the two conformers modelled, the “closed” one has the typical truncated cone shape whereas the open one is more like a cylinder (Figure 1B and C).

### Hydrated γ-CD

#### Hydration and interaction with water (sequential binding of water molecules to the CD cavity)

**Figure 2:**
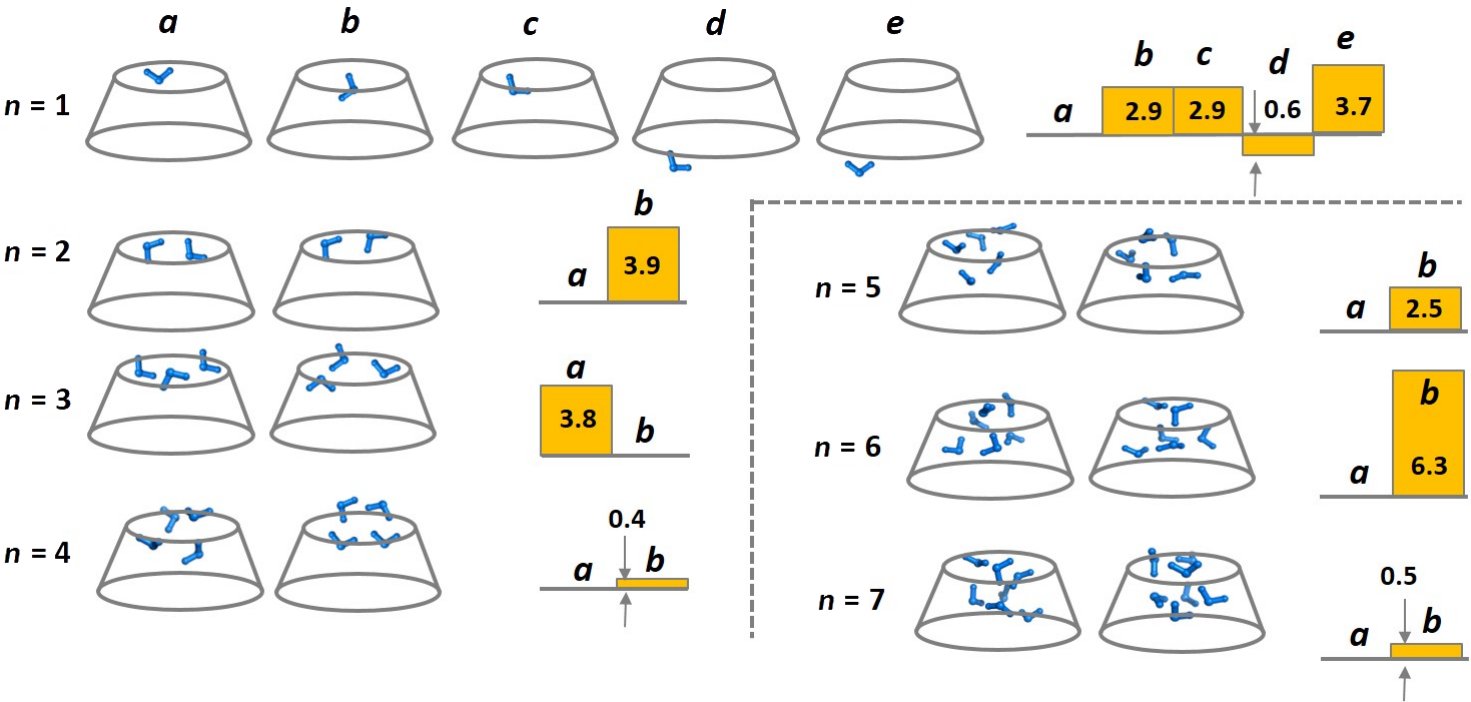
Schematic representation of γ-CD–*n*H_2_O complexes (where *n* = 1–7) with water molecules/clusters located at different positions, and M062X/6-311++G(d,p)//M062X/6-31G(d,p) calculated relative enthalpies (Δ*H*^78^) of the respective complexes, in kcal mol^−1^.

The γ-CD cavity was scanned for spots/sites with enhanced binding affinity for the incoming water molecules: γ-CD hydrates containing one to seven water molecules bound at various localities in the host molecule interior were modeled and energetically optimized (Figure 2). The hydration pattern for up to 7 water guest molecules is represented schematically in Figure 2. In the case of one water molecule, five possibilities were considered (Figure 2, *n* = 1, structures *a–e*), while for the other complexes only two options were modelled (Figure 2, *n* = 2–7, structures *a* and *b*).

The first water molecule can bind to the narrow rim (*n* = 1; Figure 2, structures *a–c*) or the wide rim (*n* = 1; Figure 2, structures *d* and *e*). All attempts to position single water molecule in the cavity or near the cavity walls resulted in one of the *a–e* variants. The enthalpies of the single water molecule constructs (γ-CD–H_2_O) are calculated relative to structure *a*, although structure *d* is energetically preferred. In structure *d*, the water molecule is completely outside the CD cavity and is bound to OH-2 and OH-3 of one monomeric glucose unit. Construct *е*, in which the water molecule binds to OH-2 and OH-3 groups from two adjacent monomers, is not as energetically favorable.

As the calculated relative enthalpies of the resulting complexes with water molecules positioned inside the CD cavity (Figure 2, structures *a–c*) suggest, the structures with a water molecule positioned at the narrow rim of γ-CD (*n* = 1; Figure 2, structures *a*–*c*) appeared to be the most stable ones. Isoenergetic structures *b* and *c* arise from initial structures with the water molecule positioned differently, in the middle of the CD cavity (*b*) and in the center of the upper rim plane. Therefore, the narrow rim with the H-bonded primary OH groups can be considered as a major attractor (anchor, hot spot) for the incoming water molecules. The identification of this hot spot location provides very useful information for modelling the γ-CD–*n*H_2_O (*n* = 2–7) constructs. Similar results had been obtained for the positioning of the first water molecule hosted by the smaller α- and β-CD counterparts [13–14].

The calculations indicate that water coordination to the narrow rim results in creating two new H-bonds between the CD host and the guest water molecule at the expense of destroying one hydrogen bond from the initial γ-CD structure. It should be noted that coordination of water to the wide rim (construct *d*) creates two new H-bonds between the CD host and the guest water molecule and one H-bond between secondary OH-groups of γ-CD, but neither of the existing H-bonds is broken.

In constructing γ-CD hydrates with *n* > 1 water molecules, each subsequent water molecule is suitably inserted into the γ-CD–(*n*−1)H_2_O complex so as to maximize its interactions with neighboring water molecules or hydroxy groups on the rims. In all cases, the *n*-th water molecule was added to form a cluster with the *n−*1 water molecules already trapped. Constructs *a* are energetically preferred over *b* for all complexes except γ-CD–3H_2_O. γ-CD–3H_2_O (*a*) has a linear cluster of three water molecules connecting opposite sites on the narrow edge, and this appears unfavorable in comparison to a single water dimer and single water molecules located on the narrow rim (γ-CD–3H_2_O (*b*)). The first layer of water molecules is filled by three water molecules in the plane of the narrow rim, and the fourth incoming guest is displaced into the cavity upon optimization (Figure 2; *n* = 4; structure *a*). Up to three layers of interconnected water molecules can be formed inside the γ-CD cavity.

**Figure 3:**
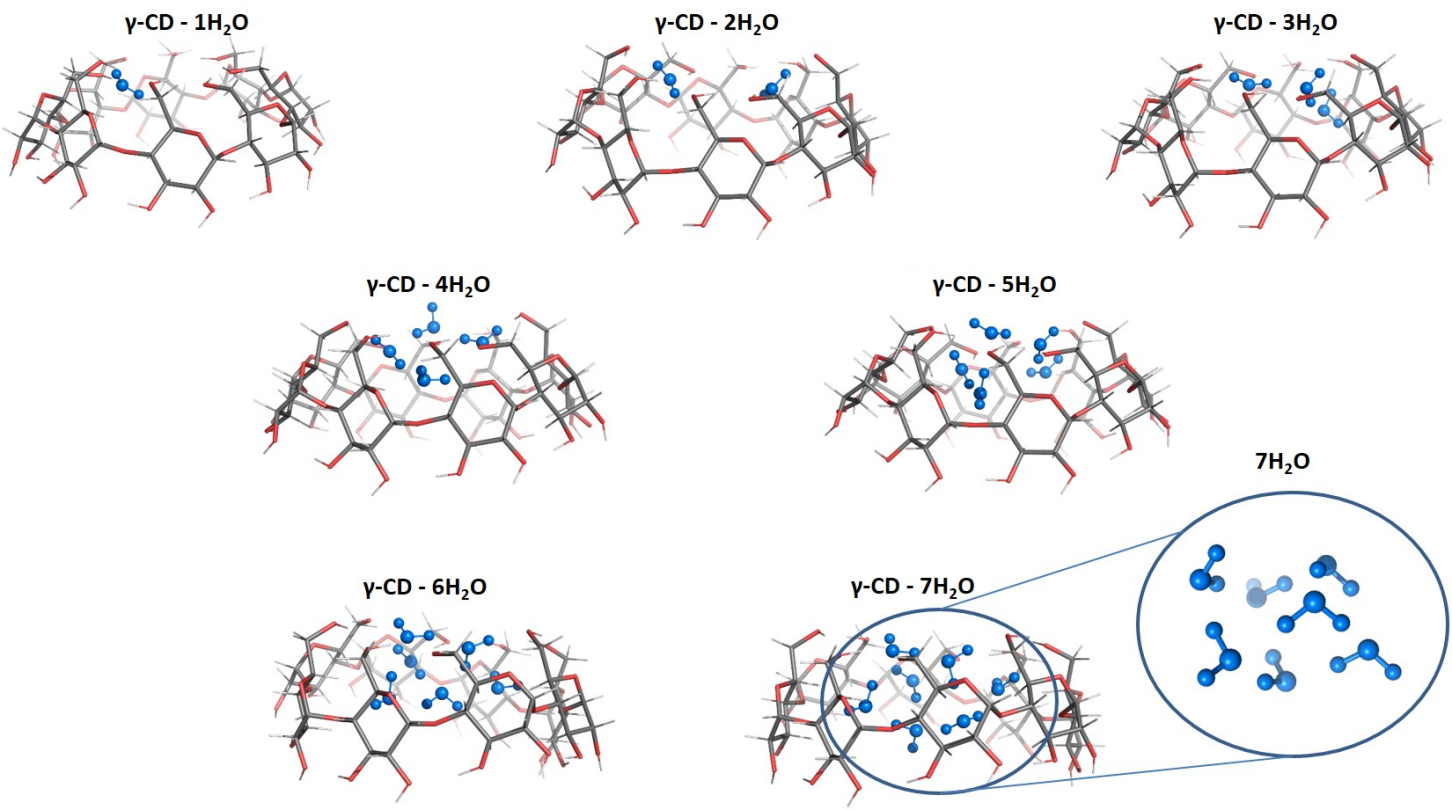
M062X/6-31G(d,p) optimized structures of the most stable (*a*/*b* structures from Figure 2) γ-CD–*n*H_2_O (*n* = 1–7) complexes with water molecules trapped in the CD cavity and not protruding out.

M062X/6-31G(d,p) optimized structures of the most stable (*a*/*b* structures from Figure 2) γ-CD–*n*H_2_O (*n* = 1–7) complexes with water molecules trapped in the CD cavity are shown in Figure 3.

The enthalpies estimated for the subsequent binding process of water molecules to γ-CD in both the gas phase (Δ*H*^1^) and aqueous medium (Δ*H*^78^) at both levels of theory are presented in Table 1. The two sets of calculations using different basis sets (M062X/6-31G(d,p) and M062X/6-311++G(d,p)//M062X/6-31G(d,p)) follow the same trend of changes. All interactions in the gas phase are favorable and characterized by a negative Δ*H*^1^. In principle, the formation of new hydrogen bonds is beneficial, while the breaking of existing ones is unfavorable to the hydration process. For this reason, the change of enthalpies is not smooth, but proceeds in steps – depending on whether the new water molecule has found a suitable location and whether the changes that occur in the dynamically mobile γ-CD host system are advantageous.

**Table 1:** M062X/6-31G(d,p) and M062X/6-311++G(d,p)//M062X/6-31G(d,p) calculated gas-phase enthalpies (Δ*H*^1^) and enthalpies in water environment (Δ*H*^78^) (in kcal mol^−1^) for the most stable γ-CD–*n*H_2_O (*n* = 1−10) complex formation.

	M062X/6-31G(d,p)		M062X/6-311++G(d,p) //M062X/6-31G(d,p)	
	Δ*H*^1^	Δ*H*^78^	Δ*H*^1^	Δ*H*^78^

1. γ-CD + H_2_O → γ-CD-H_2_O	−9.1	−4.4	−5.1	0.5
2. γ-CD-H_2_O + H_2_O → γ-CD-2H_2_O	−13.3	−8.4	−8.8	−3.7
3. γ-CD-2H_2_O + H_2_O → γ-CD-3H_2_O	−19.1	−12.3	−11.1	−5.3
4. γ-CD-3H_2_O + H_2_O → γ-CD-4H_2_O	−13.4	−7.3	−13.0	−5.0
5. γ-CD-4H_2_O + H_2_O → γ-CD-5H_2_O	−10.6	−2.2	−6.3	2.2
6. γ-CD-5H_2_O + H_2_O → γ-CD-6H_2_O	−17.4	−8.7	−13.8	−4.2
7. γ-CD-6H_2_O + H_2_O → γ-CD-7H_2_O	−13.0	−7.7	−8.1	−2.8
8. γ-CD-7H_2_O + H_2_O → γ-CD-8H_2_O	−20.2	−13.1	–	–
9. γ-CD-8H_2_O + H_2_O → γ-CD-9H_2_O	−8.8	−4.9	–	–
10. γ-CD-9H_2_O + H_2_O → γ-CD-10H_2_O	−19.4	−11.9	–	–
11. γ-CD + 7H_2_O (cluster)→ γ-CD-7H_2_O	−18.4	−12.6	−8.7	−2.1

This brings us to the main question – how many water molecules are there in the γ-CD cavity, i.e., what is the saturation point of the γ-CD internal hydration as determined by DFT modeling of possible γ-CD–*n*H_2_O complexes? In general, the gas-phase calculations at the two levels of theory (and in aqueous media at the M062X/6-31G(d,p) level) show that the sequential insertion of water molecules (up to 10) into the cavity is favorable, with no sign of the binding enthalpy reaching saturation limit. The filling of the cavity, which preferentially starts from the narrow rim, occurs by building up ‘layers’ of water molecules and the attainment of large absolute values of Δ*H* (Table 1, reactions 8 and 10) can be interpreted as the attachment of water molecules that are already located in the ε = 78 medium rather than inside the hydrophobic cavity of γ-CD. Furthermore, a close examination of the optimized structures of the γ-CD hydrates (Figure 3) reveals that the carrier cavity is quite full with 7 water molecules incorporated into it. It can be assumed that the number of H-bonds is optimal in γ-CD–7H_2_O (the complex with seven water molecules) and that when further water molecules are added, not all of them find a suitable position to form a hydrogen bond with the host molecule or with water molecules positioned adjacently. From the results for γ-CD–H_2_O complexes (with a single H_2_O molecule), we know that the water molecules associated with the wide rim of the γ-CD are actually located outside the host cavity. Thus, our results suggest (with a high degree of reliability) that the number of "true" hydrating water molecules in the γ-CD pore is about 7.

#### Effect of the addition of pre-formed water clusters (bulk binding)

The stepwise binding of water molecules to the cyclodextrin host appears to be a favorable process. As indicated in our previous studies on α- and β-CDs [13–14]**,** clusters (H_2_O)*_n_* (*n* ≥ 2) of different size and shape may exist in the aqueous phase [23], and they may also be involved in the CD hydration process. The results for the enthalpies of the hydration reaction with a water cluster of 7 molecules show that the process is advantageous (Table 1, the last row) – the *∆H*^ε^ values stay on negative ground; M062X/6-31G(d,p) calculated Δ*H*^ε^ are −18.4 kcal mol^−1^ and −12.6 kcal mol^−1^ in the gas phase and in water environment, respectively. M062X/6-311++G(d,p)//M062X/6-31G(d,p) calculated values are lower in absolute value (−8.7 kcal mol^−1^ and −2.1 kcal mol^−1^), but still negative. It should be noted that not all possible heptamers, which can be obtained with different arrangements of water molecules, were considered, but the geometry of the specific cluster that fits into the γ-CD cavity was re-optimized. The finding of a stable water cluster of 7 molecules in the cavity of the CD (with which the hydration reaction is thermodynamically favorable) is a particularly important result.

#### Thermal dehydration of γ-CD

γ-CD molecules in the crystal are stacked in such a pattern to form cage-type packing [24]. Both ends (rims) of the cyclodextrin cavity are closed by neighboring molecules, almost like in β-CD and δ-CD hydrates. According to crystallographic data the place through eight of the O atoms makes 46.5° angle with the *b* axis and as a result the two adjacent γ-CD molecules are shifted along the *b* axis with about half a molecule. In this way both ends (rims) of the γ-CD cavity are blocked, but the cavity is not entirely closed. Stacked along the *b* axis there is a narrow channel in the cavity filled with water molecules. The conclusions are that the asymmetric unit of the crystal contains 14.1 water molecules, distributed over 23 cites, while γ-CD contains 7.1 water molecules, which occupy 14 sites [24]. Literature TG data show a 7.2% mass loss up to 105 °C, with a peak maximum at 63.4 °C, corresponding to the water release [25]. DSC data correlate with the TG results, revealing an endothermic peak related to the water release at 80.9 °C [25].

**Figure 4:**
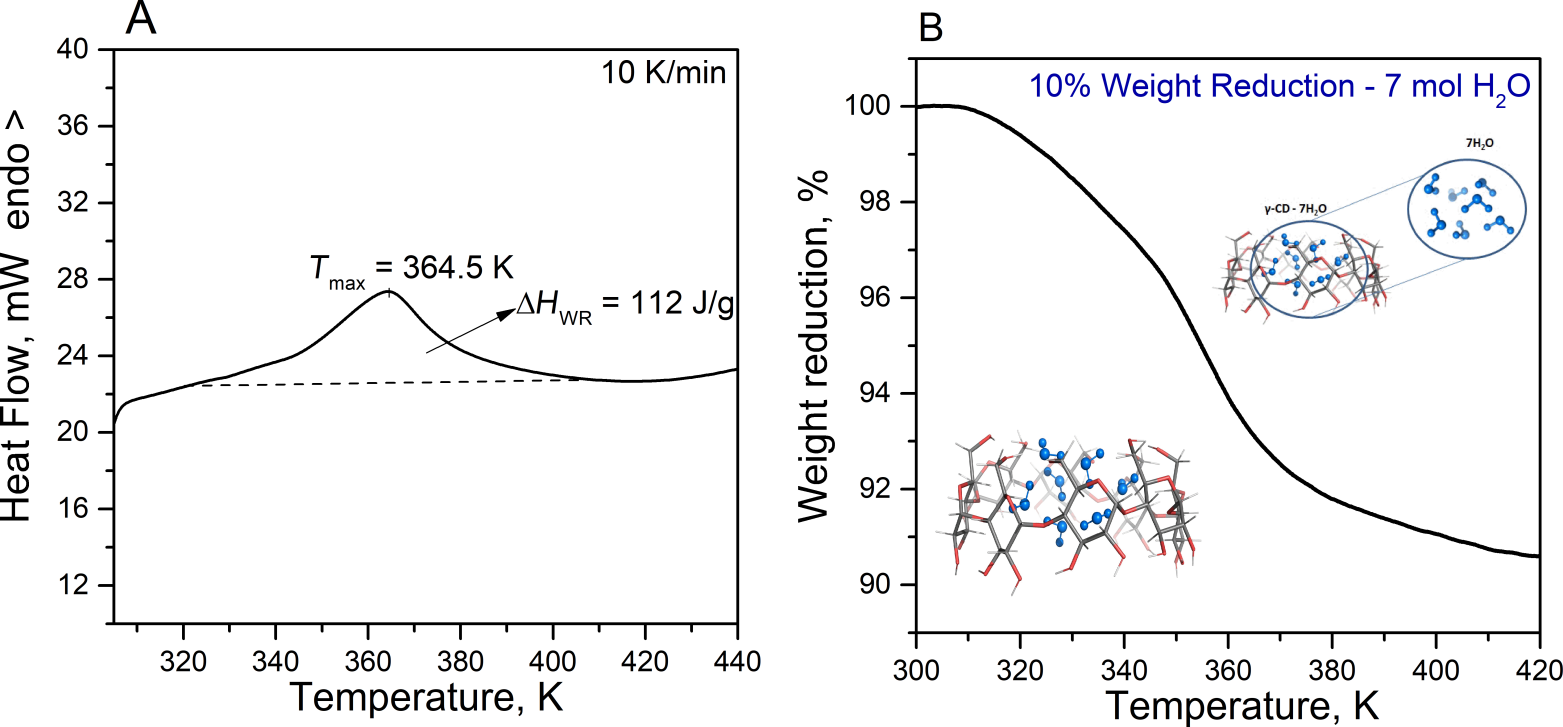
DSC curve (A) and TG analysis for γ-CD (B). Compared to our previous studies, the hydrated γ-cyclodextrin (with 7 H_2_O molecules) is placed in the middle between α-CD (with 6) and β-CD (with 10).

In the present study to measure the quantity and thermal stability of the water molecules inside the γ-CD we employed differential scanning calorimetry (DSC – Figure 4A) and thermogravimetry (TG – Figure 4B) in the temperature range between 300–440 K. The DSC curve reveals a wide endothermic effect with a maximum at about 365 K associated with the release of crystal water. The enthalpy change due to water release from γ-CD was determined to be 112 J g^−1^ (20.5 kJ mol^−1^ H_2_O). This value is lower than that (35–40 kJ mol^−1^ H_2_O) obtained for β-CD [14] and that found by Bilal et al. [26] and appears similar to the value experimentally obtained for α-CD (22.1 ± 3.8 and 30.0 ± 2.5 kJ mol^−1^ H_2_O for the first and second water release, respectively) [13]. The thermogravimetric curve shows a 10% weight loss in the temperature range 300–450 K, associated with the release of both crystal water inside the cyclodextrin cavity and in the intermolecular space. Several overlapped steps are visible on the TG curve, which is associated with the release of water molecules in several stages, with the steepest lightening of the sample at 350–370 K. Consistent with this is the DSC endothermic peak, which is asymmetric with a small shoulder at the low temperature peak side. According to the TG analysis, it was estimated that the γ-CD in the present study contains 7 mol of H_2_O. Several measurements were performed to ensure reproducibility of the results, which proved to be in line with the literature data [17,27–28].

#### Comparison between the CDs: γ-CD vs α- and β-CD

Analysis of the binding properties of CDs reveals that these very similar molecules, members of the CD family with six to eight glucose monomers in a ring (α-, β-, and γ-CD) have some common characteristics, but also differ in others. Below we list some similarities and dissimilarities that we have revealed in the theoretical (DFT) and experimental (DSC and TGA) studies of these systems, without claiming that the list is exhaustive.

#### Similarities

The hydrophobic cavities of all three family members are filled with water molecules. The process of water inclusion into CDs is energetically favorable, characterized predominantly by negative Δ*H* values. The first few incoming water molecules cluster around the narrow rim due to the higher electron density concentrated at this location. Gradually, a water cluster is formed inside the CD cavity by stepwise sequential coordination of the water guests. The resulting supramolecular architecture, composed of CD and interconnected water molecules, is stabilized by hydrogen bonds between the water molecules and CDs. The pattern previously observed for α- and β-CD hydration is fully relevant for γ-CD: the hotspot of the CD host molecule with the highest affinity for the incoming water molecule(s) is the narrow rim with **–**CH_2_OH groups [13–14]. According to the M062X/6-31G(d,p) calculations, the hydration reaction with the first incoming from the bulk water molecule (CD–H_2_O formation) is characterized by similar Δ*H*^78^ values (−2.6, −2.8, and −4.4 kcal mol^−1^ for α-, β-, and γ-CD, respectively).

#### Dissimilarities

CDs differ in both the number of water molecules that they sequester in their cavity and the manner in which the dehydration process occurs. Both experiments and theory agree that α-CD can accommodate up to six water molecules, β-CD ten water molecules, and γ-CD seven water molecules. A non-proportional relationship between the number of included water molecules and the number of monomeric CD units is observed.

There is also a significant difference between the CD dehydration processes: whereas α-CD [13] and γ-CD dehydration follows a stepwise individual water release, β-CD dehydration is characterized by a one-step release process of water content [14]. A possible explanation of this fact is the different predominance of hydrogen bonds formed between water guests (cluster formation) and between water molecules and the CD host system: β-CD hydration is dominated by water–water hydrogen bonding interactions rather than water–CD interactions. In contrast, the interactions between water guests and the cavity walls are more significant at α-CD and γ-CD, favoring a consecutive water release process upon heating.

## Conclusion

Using experimental and computational methods as well as available literature data, the present study reliably estimates the number of water molecules present in γ-CD, their preferable binding position, and thermal stability. Comparison with the other cyclodextrins revealed that in terms of the amount of water molecules, γ-CD (7 mol H_2_O) lies between α-CD (6 mol H_2_O) and β-CD (10 mol H_2_O). Furthermore, it is shown that the seven water molecules in the γ-CD form a cluster due to hydrogen bonds between the water molecules themselves as well as with the walls of the cyclodextrin host, in contrast to β-CD where their interaction with the inner walls of the CD can be neglected and therefore does not contribute to the stabilization of water complexes. As a result of this different bonding, water molecules from γ-CD are released upon annealing in 2–3 successive stages, whereas in β-CD it is a one-step process.

## Experimental

### Materials

γ-CD was used as received (without further purification or modification) from Wacker Chemie AG (CAVAMAX FOOD) with a purity of ≥98%.

### Experimental measurements

The thermal behavior of γ-CD was investigated with a Perkin Elmer DSC7 Differential Scanning Calorimeter. The samples were placed in aluminum pans and heated in the temperature range between 300–440 K at a constant rate of 10 °C min^−1^ under pure nitrogen atmosphere. Similarly, thermogravimetric measurements were conducted using DTA/TG (TA-SDT 600) at the same heating rate and atmosphere of pure nitrogen. To ensure accuracy and reproducibility of the results the measurements were repeated multiple times, and no difference was observed between individual trials.

### Computational details

A computational protocol similar to that used in our previous work on α-CD [13] and β-CD [14] hydration was employed: the geometries of γ-CD, water molecule/clusters and hydrated complexes were optimized at the M062X/6-31G(d,p) theoretical level and the electronic energy, *E*_el_, of each structure was estimated. To obtain more accurate energies, single point calculations were made at the M062X/6-311++G(d,p) level of theory using the M062X/6-31G(d,p) optimized structures. Electronic energies obtained at both levels of theory (M062X/6-31G(d,p)//M062X/6-31G(d,p) and M062X/6-311++G(d,p)//M062X/6-31G(d,p)) were used together in subsequent estimations. Validation of the method against experimental data is described in the first two papers in the series on the hydration of α-CD and β-CD, respectively [13–14]. The performed M062X/6-31G(d,p) frequency calculations for each structure ascertain that the wave function corresponds to a minimum on a potential energy hypersurface, but also yields the thermochemistry (and thermodynamic values). To assess the thermodynamic feasibility of the complex formation reaction, we used the enthalpy change, Δ*H*, when going from reagents to products.

The SMD [29] model is used to incorporate the effects of water as a solvent in the molecular simulations – single point calculations were carried out at both levels of theory.

All the calculations in the gas phase and in water environment were carried out with the Gaussian 09 suite of programs [30]. Basis set superposition error (BSSE) was computed using the counterpoise procedure of Boys and Bernardi [31] implemented in the G09 package. The PyMOL software was used to create molecular graphics images [32].

## Supporting Information

File 1 Additional figures and table with optimized geometries for γ-CD.

## Data Availability

The data that supports the findings of this study is available from the corresponding author upon reasonable request.
