# Supplementary material for: Deciphering the mechanism of γ-cyclodextrin’s hydrophobic cavity hydration: an integrated experimental and theoretical study
Source: Beilstein J Org Chem. 2024 Oct 17;20:2635–43. doi: 10.3762/bjoc.20.221 (PMC11496703; doi:10.3762/bjoc.20.221)
Supplement: File 1 — Additional figures and table with optimized geometries for γ-CD. [file Beilstein_J_Org_Chem-20-2635-s001.pdf]

BEILSTEIN JOURNAL OF ORGANIC CHEMISTRY

Supporting Information

for

Deciphering the mechanism of γ-cyclodextrin's hydrophobic cavity hydration: an integrated experimental and theoretical study

Stiliyana Pereva, Stefan Dobrev, Tsveta Sarafska, Valya Nikolova, Silvia Angelova, Tony Spassov and Todor Dudev

Beilstein J. Org. Chem. **2024,** *20,* 2635–2643. doi:10.3762/bjoc.20.221

Additional figures and table with optimized geometries for γ-CD

License and Terms: This is a supporting information file under the terms of the Creative Commons Attribution License (https://creativecommons.org/licenses/by/4.0). Please note that the reuse, redistribution and reproduction in particular requires that the author(s) and source are credited and that individual graphics may be subject to special legal provisions.
The license is subject to the *Beilstein Journal of Organic Chemistry* terms and conditions: (https://www.beilstein-journals.org/bjoc/terms)

Table of contents:

Figure S1. Schematic representation of γ-CD–H_2O complexes with water molecule located at different positions, and M062X/6-311++G(d,p)//M062X/6-31G(d,p) calculated relative enthalpies (ΔH^{78}) of the respective complexes, in kcal mol^{-1}. γ-CD–H_2O (*f*) is a complex in which a single water molecule is located outside the CD cavity, near the oxygen atom of one of the glucopyranose units...S2

Figure S2. H-bonds in γ-CD–H_2O (*a*) and γ-CD–H_2O (*d*) complexes. S2

Table S1. M062X/6-31G(d,p) optimized geometries of γ-CD, selected γ-CD–nH_2O (n = 1−7) complexes, and a cluster of 7 water molecules. S3

Figure S1. Schematic representation of γ-CD–H_2O complexes with water molecule located at different positions, and M062X/6-311++G(d,p)//M062X/6-31G(d,p) calculated relative enthalpies (ΔH^{78}) of the respective complexes, in kcal mol^{-1}. γ-CD-H_2O (*f*) is a complex in which a single water molecule is located outside the CD cavity, near the oxygen atom of one of the glucopyranose units.

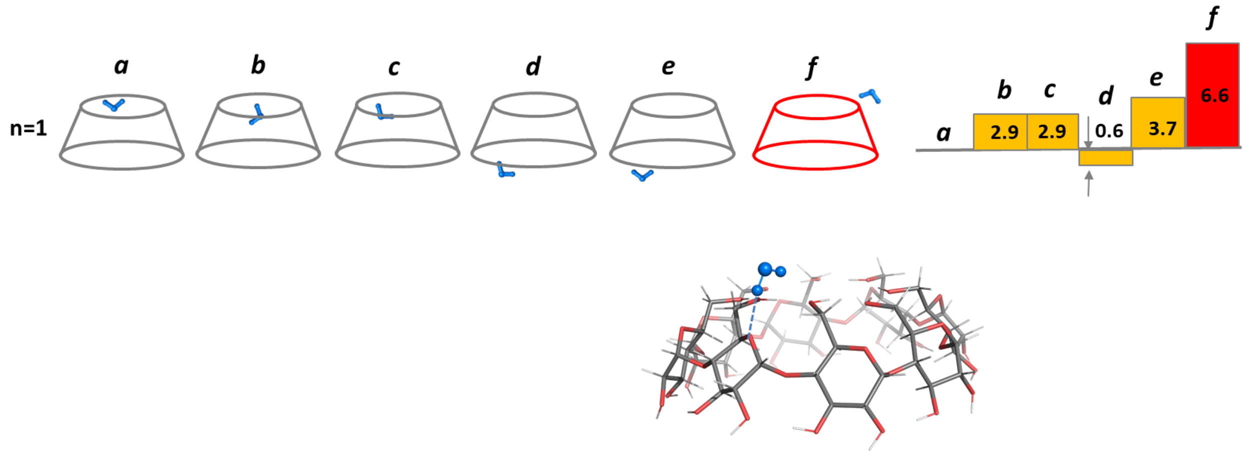

Figure S2. H-bonds in γ-CD–H_2O (*a*) and γ-CD–H_2O (*d*) complexes.

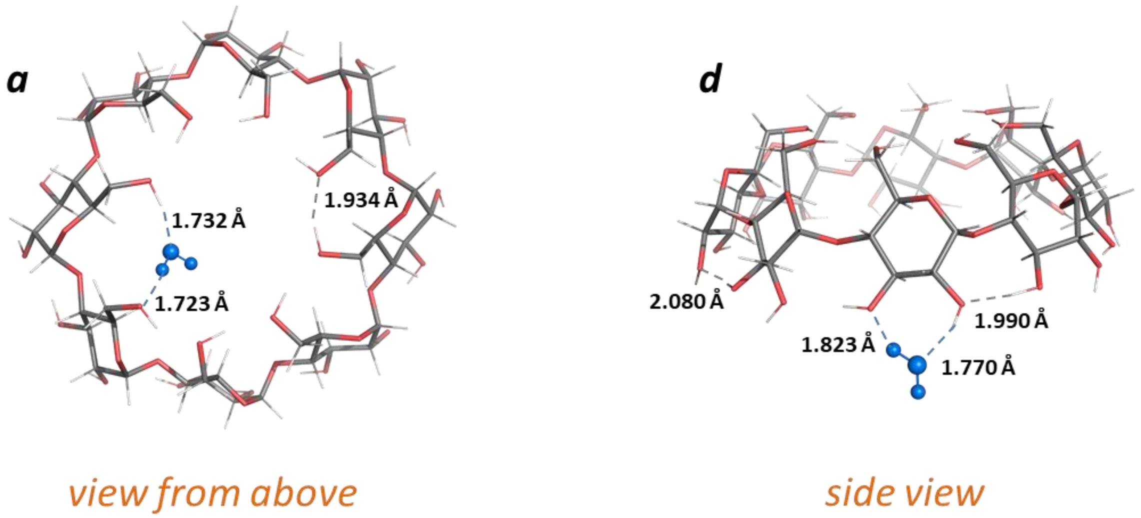

Table S1. M062X/6-31G(d,p) optimized geometries of γ-CD, selected γ-CD–nH_2O (n = 1–7) complexes, and a cluster of 7 water molecules.

**γ-CD**			
6	1.065743000	-6.749738000	0.139061000
6	0.197080000	-7.342359000	-0.969302000
6	-0.966496000	-6.424075000	-1.291448000
6	-1.684189000	-6.050316000	-0.003626000
6	-0.690933000	-5.392729000	0.954952000
6	-1.269603000	-4.871614000	2.275235000
8	1.736368000	-5.637251000	-0.375387000
8	1.009378000	-7.571892000	-2.101331000
8	-1.805098000	-7.112188000	-2.184971000
8	0.282994000	-6.403875000	1.253388000
8	-1.177108000	-3.461214000	2.362789000
1	-2.068260000	-6.964735000	0.473576000
1	1.782808000	-7.498511000	0.494820000
1	-0.219607000	-8.288697000	-0.593231000
1	-0.570470000	-5.499009000	-1.741899000
1	-0.198377000	-4.548157000	0.455466000
1	-2.329243000	-5.122882000	2.367863000
1	-0.720384000	-5.353350000	3.094392000
1	0.408773000	-7.792827000	-2.826360000
1	-2.634761000	-6.610580000	-2.275964000
1	-0.224891000	-3.247583000	2.407736000
6	-4.021894000	-5.528366000	0.130327000
6	-5.051683000	-5.328471000	-0.980630000
6	-5.226142000	-3.854905000	-1.296093000
6	-5.472129000	-3.089673000	-0.004877000
6	-4.306957000	-3.330994000	0.955638000
6	-4.351480000	-2.559184000	2.279392000
8	-2.758907000	-5.212115000	-0.377076000
8	-4.635404000	-6.060613000	-2.114132000
8	-6.305697000	-3.746116000	-2.189556000
8	-4.334341000	-4.735982000	1.247709000
8	-3.292422000	-1.623723000	2.372744000
1	-6.390899000	-3.467765000	0.468883000
1	-4.044759000	-6.566706000	0.480685000
1	-6.016516000	-5.705185000	-0.609277000
1	-4.292406000	-3.476065000	-1.743648000
1	-3.359835000	-3.080803000	0.459970000
1	-5.280652000	-1.991548000	2.373470000
1	-4.301959000	-3.291681000	3.095463000
1	-5.215743000	-5.792291000	-2.839564000
1	-6.543486000	-2.805424000	-2.272164000
1	-2.464898000	-2.141474000	2.412714000
6	-6.752577000	-1.065444000	0.141203000
6	-7.346179000	-0.197107000	-0.966808000
6	-6.427960000	0.966232000	-1.290189000
6	-6.052578000	1.684090000	-0.002900000
6	-5.394523000	0.690827000	0.955521000
6	-4.872119000	1.269303000	2.275308000

8	-5.640475000	-1.736065000	-0.373992000
8	-7.576915000	-1.009697000	-2.098386000
8	-7.116970000	1.804742000	-2.183086000
8	-6.405829000	-0.282480000	1.255061000
8	-3.461544000	1.177322000	2.360904000
1	-6.966244000	2.068609000	0.475387000
1	-7.500993000	-1.782465000	0.497805000
1	-8.292079000	0.219826000	-0.589897000
1	-5.503507000	0.569833000	-1.741564000
1	-4.550655000	0.197699000	0.455388000
1	-5.123966000	2.328738000	2.368704000
1	-5.352297000	0.719444000	3.094926000
1	-7.798092000	-0.409213000	-2.823447000
1	-6.614958000	2.634008000	-2.275534000
1	-3.247749000	0.225140000	2.405950000
6	-5.529786000	4.021725000	0.130244000
6	-5.329258000	5.050917000	-0.981148000
6	-3.855579000	5.223768000	-1.297057000
6	-3.090197000	5.470019000	-0.005979000
6	-3.332078000	4.305455000	0.955199000
6	-2.559778000	4.350105000	2.278639000
8	-5.214509000	2.758562000	-0.377398000
8	-6.062100000	4.634813000	-2.114266000
8	-3.745888000	6.302569000	-2.191313000
8	-4.737051000	4.334078000	1.247480000
8	-1.623784000	3.291579000	2.371221000
1	-3.468258000	6.389043000	0.467320000
1	-6.568085000	4.045308000	0.480700000
1	-5.704902000	6.016308000	-0.610170000
1	-3.477675000	4.289370000	-1.744012000
1	-3.082798000	3.357781000	0.460120000
1	-1.992603000	5.279561000	2.372719000
1	-3.291869000	4.300062000	3.095067000
1	-5.793366000	5.214452000	-2.840101000
1	-2.805038000	6.539771000	-2.273788000
1	-2.141347000	2.463937000	2.410963000
6	-1.065663000	6.750224000	0.139978000
6	-0.196922000	7.343415000	-0.968012000
6	0.966732000	6.425370000	-1.290637000
6	1.684187000	6.050550000	-0.002995000
6	0.690625000	5.392262000	0.954774000
6	1.268603000	4.869645000	2.274719000
8	-1.736552000	5.638197000	-0.375132000
8	-1.009127000	7.573618000	-2.099968000
8	1.805520000	7.114162000	-2.183463000
8	-0.283012000	6.403358000	1.254065000
8	1.177259000	3.459085000	2.359770000
1	2.068125000	6.964506000	0.475194000
1	-1.782441000	7.498961000	0.496399000
1	0.219679000	8.289541000	-0.591311000
1	0.570769000	5.500639000	-1.741842000
1	0.197789000	4.548435000	0.454301000
1	2.327917000	5.121794000	2.368792000

1	0.718166000	5.349489000	3.094158000
1	-0.408449000	7.795062000	-2.824778000
1	2.635164000	6.612565000	-2.274723000
1	0.225124000	3.244779000	2.403360000
6	4.022004000	5.528488000	0.130868000
6	5.051980000	5.328541000	-0.979924000
6	5.226531000	3.855039000	-1.295764000
6	5.471902000	3.089638000	-0.004615000
6	4.306009000	3.330822000	0.955014000
6	4.348754000	2.557482000	2.277906000
8	2.759069000	5.212787000	-0.377044000
8	4.636054000	6.060968000	-2.113374000
8	6.306379000	3.746619000	-2.188935000
8	4.333929000	4.735618000	1.248188000
8	3.289056000	1.622733000	2.369161000
1	6.390221000	3.467774000	0.469974000
1	4.045136000	6.566736000	0.481467000
1	6.016768000	5.705054000	-0.608248000
1	4.293001000	3.476276000	-1.743810000
1	3.359223000	3.081791000	0.458140000
1	5.277439000	1.989070000	2.372072000
1	4.299166000	3.289032000	3.094839000
1	5.216560000	5.792760000	-2.838713000
1	6.544041000	2.805950000	-2.272093000
1	2.461840000	2.141100000	2.408403000
6	6.752904000	1.065567000	0.141650000
6	7.346719000	0.196805000	-0.965970000
6	6.428431000	-0.966357000	-1.289544000
6	6.052711000	-1.684027000	-0.002280000
6	5.394644000	-0.690825000	0.955934000
6	4.872161000	-1.270316000	2.275325000
8	5.640906000	1.736069000	-0.373873000
8	7.578177000	1.009096000	-2.097623000
8	7.117522000	-1.805009000	-2.182258000
8	6.406006000	0.282606000	1.255603000
8	3.461627000	-1.177990000	2.361441000
1	6.966291000	-2.068642000	0.476100000
1	7.501261000	1.782669000	0.498201000
1	8.292351000	-0.220327000	-0.588606000
1	5.504163000	-0.569835000	-1.741183000
1	4.550816000	-0.197627000	0.455834000
1	5.123446000	-2.329997000	2.367676000
1	5.352770000	-0.721337000	3.095305000
1	7.799515000	0.408378000	-2.822437000
1	6.615364000	-2.634153000	-2.275010000
1	3.247952000	-0.225764000	2.406601000
6	5.529880000	-4.021679000	0.129707000
6	5.328934000	-5.050154000	-0.982222000
6	3.855137000	-5.222537000	-1.297687000
6	3.090183000	-5.469640000	-0.006537000
6	3.332545000	-4.305925000	0.955532000
6	2.561074000	-4.351858000	2.279420000
8	5.214148000	-2.758147000	-0.376498000

8	6.061319000	-4.633507000	-2.115432000
8	3.745042000	-6.300570000	-2.192824000
8	4.737676000	-4.334621000	1.247130000
8	1.625749000	-3.292819000	2.374041000
1	3.468144000	-6.389149000	0.465905000
1	6.568364000	-4.045520000	0.479587000
1	5.704659000	-6.015781000	-0.611943000
1	3.477212000	-4.287717000	-1.743744000
1	3.082979000	-3.357779000	0.461500000
1	1.993374000	-5.281063000	2.372787000
1	3.293788000	-4.303192000	3.095366000
1	5.792333000	-5.212859000	-2.841406000
1	2.804174000	-6.537791000	-2.275092000
1	2.143660000	-2.465409000	2.414698000
**γ-CD-H_2O(a)**			
6	6.615442000	1.871140000	0.207943000
6	7.383801000	0.998953000	-0.781648000
6	6.650742000	-0.307177000	-0.996670000
6	6.383035000	-0.982693000	0.336513000
6	5.655612000	-0.026547000	1.284688000
6	5.508717000	-0.589011000	2.686507000
8	5.402165000	2.244773000	-0.383473000
8	7.520545000	1.706419000	-1.997344000
8	7.445419000	-1.100106000	-1.841240000
8	6.411739000	1.182904000	1.411800000
8	4.520227000	0.081432000	3.447250000
1	7.340559000	-1.297263000	0.780796000
1	7.204581000	2.758928000	0.464957000
1	8.370768000	0.780670000	-0.347953000
1	5.673739000	-0.083863000	-1.455652000
1	4.661629000	0.192540000	0.873119000
1	5.205276000	-1.638450000	2.625953000
1	6.487726000	-0.532311000	3.182795000
1	7.841871000	1.062614000	-2.644846000
1	7.081384000	-2.003924000	-1.847115000
1	4.546178000	1.038413000	3.255105000
6	5.882832000	-3.343459000	0.608965000
6	5.908374000	-4.394446000	-0.502014000
6	4.513534000	-4.603788000	-1.061544000
6	3.557064000	-4.886188000	0.085357000
6	3.589010000	-3.722106000	1.072957000
6	2.633415000	-3.851410000	2.259399000
8	5.587026000	-2.110090000	0.023087000
8	6.808595000	-3.973515000	-1.506970000
8	4.588824000	-5.675222000	-1.969510000
8	4.930213000	-3.682082000	1.585220000
8	1.426952000	-3.157170000	2.014949000
1	3.899984000	-5.780987000	0.626761000
1	6.848959000	-3.304891000	1.126161000
1	6.242528000	-5.344814000	-0.060433000
1	4.191232000	-3.675523000	-1.560443000
1	3.365825000	-2.774658000	0.566081000

1	2.370679000	-4.898944000	2.439744000
1	3.146683000	-3.459781000	3.148249000
1	6.663361000	-4.565948000	-2.258168000
1	3.691161000	-5.871280000	-2.292513000
1	1.645995000	-2.200746000	1.973076000
6	1.732919000	-6.385105000	-0.232443000
6	1.006101000	-6.858269000	-1.491184000
6	-0.264141000	-6.062543000	-1.721658000
6	-1.086714000	-6.063678000	-0.443653000
6	-0.249752000	-5.473624000	0.690021000
6	-0.959814000	-5.329909000	2.041809000
8	2.281780000	-5.122098000	-0.476279000
8	1.892752000	-6.761393000	-2.586021000
8	-0.947860000	-6.663783000	-2.793068000
8	0.866407000	-6.359379000	0.870877000
8	-1.132238000	-3.972358000	2.403053000
1	-1.351106000	-7.100479000	-0.185191000
1	2.523320000	-7.100538000	0.022826000
1	0.713368000	-7.906069000	-1.324217000
1	0.007421000	-5.019302000	-1.952408000
1	0.120004000	-4.483600000	0.395967000
1	-1.956435000	-5.776828000	2.018405000
1	-0.355405000	-5.860710000	2.789382000
1	1.363424000	-6.905233000	-3.382105000
1	-1.848747000	-6.295852000	-2.823625000
1	-0.235403000	-3.579851000	2.420799000
6	-3.479806000	-5.867193000	-0.330914000
6	-4.498702000	-5.637617000	-1.447659000
6	-4.877165000	-4.171086000	-1.548678000
6	-5.251277000	-3.647487000	-0.171021000
6	-4.083933000	-3.874494000	0.789369000
6	-4.251613000	-3.319070000	2.208977000
8	-2.257113000	-5.322238000	-0.729841000
8	-3.950606000	-6.120510000	-2.656401000
8	-5.946738000	-4.073552000	-2.456202000
8	-3.926790000	-5.297781000	0.872930000
8	-3.319563000	-2.286757000	2.477801000
1	-6.123730000	-4.206539000	0.201192000
1	-3.374086000	-6.940831000	-0.135635000
1	-5.408888000	-6.196499000	-1.183490000
1	-3.998869000	-3.604622000	-1.900197000
1	-3.171003000	-3.431267000	0.371213000
1	-5.245096000	-2.892954000	2.359287000
1	-4.115477000	-4.153918000	2.908076000
1	-4.553905000	-5.844462000	-3.359600000
1	-6.354327000	-3.195429000	-2.351870000
1	-2.454002000	-2.737900000	2.512800000
6	-6.710622000	-1.804209000	0.328828000
6	-7.518484000	-0.929921000	-0.629300000
6	-6.784498000	0.363668000	-0.927968000
6	-6.338117000	1.013957000	0.372272000
6	-5.473881000	0.030022000	1.159894000
6	-4.860181000	0.555845000	2.462314000

8	-5.593612000	-2.288297000	-0.356342000
8	-7.765747000	-1.671607000	-1.805351000
8	-7.662973000	1.185646000	-1.655033000
8	-6.334430000	-1.074510000	1.468518000
8	-3.442832000	0.547806000	2.408157000
1	-7.223328000	1.264449000	0.976364000
1	-7.327012000	-2.635566000	0.690712000
1	-8.462109000	-0.669888000	-0.126544000
1	-5.880081000	0.125861000	-1.512293000
1	-4.646783000	-0.316478000	0.526954000
1	-5.160839000	1.588495000	2.653590000
1	-5.221079000	-0.078133000	3.281548000
1	-8.159686000	-1.055033000	-2.437612000
1	-7.278025000	2.078796000	-1.701362000
1	-3.192088000	-0.394808000	2.409667000
6	-5.973078000	3.371832000	0.675342000
6	-6.042086000	4.500227000	-0.351731000
6	-4.660082000	4.791171000	-0.903572000
6	-3.687252000	4.992013000	0.248684000
6	-3.687022000	3.757497000	1.149622000
6	-2.709604000	3.798011000	2.330543000
8	-5.656373000	2.193809000	-0.002718000
8	-6.942541000	4.123640000	-1.371832000
8	-4.765402000	5.937383000	-1.710749000
8	-5.021017000	3.662470000	1.668598000
8	-1.685940000	2.824530000	2.202754000
1	-4.016078000	5.850515000	0.853490000
1	-6.932564000	3.271673000	1.195990000
1	-6.396700000	5.404286000	0.165614000
1	-4.324583000	3.917695000	-1.486149000
1	-3.457881000	2.861586000	0.558324000
1	-2.212109000	4.770400000	2.389396000
1	-3.287770000	3.642239000	3.250276000
1	-6.836728000	4.774446000	-2.079435000
1	-3.873068000	6.188942000	-2.008032000
1	-2.121231000	1.954360000	2.280942000
6	-1.803928000	6.452793000	0.048336000
6	-1.121627000	7.047497000	-1.182985000
6	0.051057000	6.183759000	-1.600933000
6	0.941909000	5.949889000	-0.391026000
6	0.135083000	5.275105000	0.718541000
6	0.924729000	4.910420000	1.979638000
8	-2.428412000	5.264207000	-0.333339000
8	-2.080935000	7.168747000	-2.211344000
8	0.729037000	6.862302000	-2.629570000
8	-0.876772000	6.225712000	1.081833000
8	1.104021000	3.508390000	2.103038000
1	1.283237000	6.920112000	-0.000380000
1	-2.535915000	7.162184000	0.450300000
1	-0.728015000	8.035884000	-0.901792000
1	-0.331177000	5.209274000	-1.944789000
1	-0.341181000	4.358911000	0.344131000
1	1.923706000	5.357006000	1.951442000

1	0.385311000	5.317887000	2.844470000
1	-1.590840000	7.372352000	-3.019683000
1	1.514601000	6.343245000	-2.877510000
1	0.216389000	3.118495000	2.221941000
6	3.297184000	5.806344000	-0.692863000
6	4.173040000	5.398000000	-1.872804000
6	4.543508000	3.925912000	-1.802723000
6	5.090211000	3.622879000	-0.416803000
6	4.059366000	4.025586000	0.634311000
6	4.427482000	3.683147000	2.077542000
8	2.048874000	5.193955000	-0.834939000
8	3.482446000	5.712061000	-3.063448000
8	5.487192000	3.690409000	-2.816211000
8	3.897932000	5.444088000	0.526166000
8	3.745134000	2.523432000	2.511446000
1	6.001586000	4.219968000	-0.258267000
1	3.190380000	6.896672000	-0.668570000
1	5.107684000	5.974446000	-1.803055000
1	3.632409000	3.323151000	-1.952561000
1	3.101669000	3.533681000	0.418350000
1	5.495756000	3.473495000	2.184680000
1	4.184671000	4.550397000	2.705466000
1	3.991010000	5.326514000	-3.789466000
1	5.980487000	2.879002000	-2.600143000
1	2.795197000	2.744849000	2.549790000
8	2.446392000	-0.666134000	1.915095000
1	2.150512000	0.140187000	1.481152000
1	3.090145000	-0.380509000	2.603520000
**γ-CD-2H_2O(a)**			
6	6.961810000	-0.651159000	-0.246784000
6	7.535407000	0.412567000	0.686503000
6	6.545503000	1.545437000	0.854323000
6	6.116382000	2.072981000	-0.503631000
6	5.597295000	0.933649000	-1.381972000
6	5.290388000	1.380042000	-2.799728000
8	5.862234000	-1.241473000	0.386949000
8	7.825820000	-0.191885000	1.930901000
8	7.169262000	2.534249000	1.634213000
8	6.601376000	-0.082359000	-1.475867000
8	4.453764000	0.472581000	-3.490476000
1	6.977331000	2.559179000	-0.989096000
1	7.724728000	-1.405249000	-0.473273000
1	8.450862000	0.813255000	0.226992000
1	5.647397000	1.146225000	1.353574000
1	4.686292000	0.522150000	-0.927753000
1	4.759128000	2.336078000	-2.769009000
1	6.242965000	1.519902000	-3.330564000
1	8.032131000	0.532548000	2.538408000
1	6.649487000	3.355236000	1.564919000
1	4.729905000	-0.444195000	-3.289284000
6	5.091268000	4.232185000	-0.924847000
6	5.004100000	5.363325000	0.101485000

6	3.652742000	5.341277000	0.789088000
6	2.559643000	5.324534000	-0.266374000
6	2.733967000	4.111486000	-1.176511000
6	1.671606000	3.972252000	-2.271215000
8	5.102901000	3.021972000	-0.226449000
8	6.057399000	5.213813000	1.031949000
8	3.581863000	6.483954000	1.606506000
8	4.006958000	4.290402000	-1.816931000
8	0.671199000	3.038660000	-1.908498000
1	2.658582000	6.219463000	-0.899257000
1	5.995143000	4.335027000	-1.537038000
1	5.097792000	6.318864000	-0.435314000
1	3.575449000	4.416215000	1.382753000
1	2.746803000	3.180614000	-0.595691000
1	1.169213000	4.929016000	-2.448225000
1	2.182638000	3.667139000	-3.193901000
1	5.870286000	5.838738000	1.746752000
1	2.705845000	6.506558000	2.030932000
1	1.102631000	2.157247000	-1.903315000
6	0.527183000	6.477176000	0.176126000
6	-0.180940000	6.858179000	1.475755000
6	-1.236776000	5.830974000	1.832428000
6	-2.134210000	5.612345000	0.624882000
6	-1.285869000	5.147667000	-0.556533000
6	-2.060812000	4.807203000	-1.832377000
8	1.320635000	5.351308000	0.413226000
8	0.790507000	6.992716000	2.491369000
8	-1.951284000	6.323644000	2.939427000
8	-0.395781000	6.233749000	-0.854510000
8	-2.065776000	3.410809000	-2.081254000
1	-2.610542000	6.564647000	0.346687000
1	1.144703000	7.316477000	-0.163531000
1	-0.693007000	7.816827000	1.302311000
1	-0.737623000	4.877034000	2.067755000
1	-0.698944000	4.264486000	-0.275389000
1	-3.106594000	5.119467000	-1.750498000
1	-1.596676000	5.354628000	-2.662963000
1	0.308365000	7.060951000	3.326492000
1	-2.677593000	5.705396000	3.134163000
1	-1.129211000	3.141285000	-2.171919000
6	-4.447688000	5.091468000	0.917944000
6	-5.226766000	4.469431000	2.071797000
6	-5.339796000	2.964188000	1.902927000
6	-5.844745000	2.650644000	0.503317000
6	-4.941054000	3.308064000	-0.535125000
6	-5.309797000	3.023555000	-1.993160000
8	-3.117747000	4.673470000	1.011553000
8	-4.580359000	4.815679000	3.278617000
8	-6.212500000	2.499524000	2.902043000
8	-5.012332000	4.721758000	-0.316205000
8	-4.443931000	2.063716000	-2.560127000
1	-6.863568000	3.054201000	0.395410000
1	-4.512619000	6.184435000	0.967751000

1	-6.245166000	4.885043000	2.043518000
1	-4.335509000	2.522244000	2.009111000
1	-3.907882000	2.968060000	-0.387762000
1	-6.323389000	2.622271000	-2.084149000
1	-5.269420000	3.973857000	-2.542114000
1	-4.997370000	4.292125000	3.976052000
1	-6.548927000	1.624720000	2.637849000
1	-3.549417000	2.456864000	-2.574377000
6	-6.962012000	0.651317000	-0.247034000
6	-7.535852000	-0.412533000	0.685957000
6	-6.545974000	-1.545406000	0.853926000
6	-6.116321000	-2.072693000	-0.503965000
6	-5.596935000	-0.933236000	-1.381918000
6	-5.289419000	-1.379167000	-2.799700000
8	-5.862735000	1.241620000	0.387185000
8	-7.826612000	0.191831000	1.930318000
8	-7.170004000	-2.534381000	1.633385000
8	-6.601045000	0.082771000	-1.476048000
8	-4.452202000	-0.471448000	-3.489530000
1	-6.977056000	-2.558875000	-0.989813000
1	-7.724935000	1.405370000	-0.473672000
1	-8.451168000	-0.813219000	0.226167000
1	-5.648068000	-1.146251000	1.353574000
1	-4.686144000	-0.521728000	-0.927274000
1	-4.758258000	-2.335261000	-2.769200000
1	-6.241703000	-1.518531000	-3.331137000
1	-8.033175000	-0.532638000	2.537699000
1	-6.649927000	-3.355220000	1.564540000
1	-4.729286000	0.445234000	-3.289196000
6	-5.091384000	-4.232066000	-0.924626000
6	-5.004104000	-5.362922000	0.102000000
6	-3.652634000	-5.340790000	0.789408000
6	-2.559734000	-5.324501000	-0.266269000
6	-2.734107000	-4.111689000	-1.176694000
6	-1.672004000	-3.972834000	-2.271627000
8	-5.102872000	-3.021670000	-0.226551000
8	-6.057228000	-5.213150000	1.032617000
8	-3.581823000	-6.483207000	1.607170000
8	-4.007236000	-4.290618000	-1.816875000
8	-0.670938000	-3.040009000	-1.908852000
1	-2.658931000	-6.219603000	-0.898862000
1	-5.995365000	-4.335050000	-1.536635000
1	-5.097915000	-6.318595000	-0.434538000
1	-3.575123000	-4.415545000	1.382760000
1	-2.746771000	-3.180648000	-0.596135000
1	-1.170250000	-4.929846000	-2.449137000
1	-2.183114000	-3.667027000	-3.194035000
1	-5.869888000	-5.837782000	1.747617000
1	-2.705690000	-6.505940000	2.031355000
1	-1.101761000	-2.158249000	-1.903887000
6	-0.527159000	-6.477148000	0.175876000
6	0.180884000	-6.858353000	1.475503000
6	1.236874000	-5.831353000	1.832214000

6	2.134486000	-5.612910000	0.624749000
6	1.286329000	-5.147991000	-0.556705000
6	2.061400000	-4.807825000	-1.832581000
8	-1.320533000	-5.351214000	0.413005000
8	-0.790548000	-6.992768000	2.491153000
8	1.951196000	-6.324044000	2.939321000
8	0.395857000	-6.233770000	-0.854710000
8	2.065800000	-3.411583000	-2.082355000
1	2.610798000	-6.565258000	0.346675000
1	-1.144761000	-7.316346000	-0.163877000
1	0.692769000	-7.817084000	1.301996000
1	0.737879000	-4.877297000	2.067413000
1	0.699727000	-4.264615000	-0.275488000
1	3.107297000	-5.119555000	-1.750228000
1	1.597744000	-5.355983000	-2.662954000
1	-0.308391000	-7.061166000	3.326254000
1	2.677615000	-5.705919000	3.134039000
1	1.129092000	-3.142390000	-2.172708000
6	4.448010000	-5.091788000	0.917549000
6	5.227180000	-4.469782000	2.071355000
6	5.339961000	-2.964444000	1.902801000
6	5.844326000	-2.650517000	0.503053000
6	4.940298000	-3.307884000	-0.535080000
6	5.307856000	-3.022427000	-1.993177000
8	3.118028000	-4.674136000	1.011679000
8	4.581032000	-4.816387000	3.278203000
8	6.213052000	-2.500063000	2.901713000
8	5.012320000	-4.721605000	-0.316650000
8	4.442305000	-2.060939000	-2.557909000
1	6.863095000	-3.054049000	0.394632000
1	4.513217000	-6.184754000	0.967056000
1	6.245660000	-4.885170000	2.042806000
1	4.335695000	-2.522598000	2.009521000
1	3.907043000	-2.968450000	-0.387067000
1	6.321849000	-2.622322000	-2.084971000
1	5.265608000	-3.972073000	-2.543109000
1	4.998122000	-4.292947000	3.975677000
1	6.549146000	-1.625046000	2.637802000
1	3.548050000	-2.454607000	-2.574037000
8	2.281309000	0.849350000	-1.926472000
1	2.195302000	0.013541000	-1.456572000
1	2.954378000	0.685572000	-2.625999000
8	-2.280616000	-0.851225000	-1.926486000
1	-2.191327000	-0.015840000	-1.456541000
1	-2.953875000	-0.685607000	-2.625501000
**γ-CD–3H_2O(b)**			
6	-5.686416000	-3.679007000	-0.423323000
6	-5.504876000	-4.915445000	0.454855000
6	-4.040539000	-5.158020000	0.761762000
6	-3.237285000	-5.152809000	-0.529767000
6	-3.467713000	-3.828989000	-1.257110000
6	-2.726290000	-3.691632000	-2.571568000

8	-5.399968000	-2.546398000	0.343328000
8	-6.251681000	-4.730473000	1.639991000
8	-3.965187000	-6.385920000	1.439382000
8	-4.866252000	-3.740182000	-1.556952000
8	-2.676560000	-2.352944000	-3.010243000
1	-3.572607000	-5.982182000	-1.171125000
1	-6.717436000	-3.640070000	-0.793568000
1	-5.877314000	-5.785150000	-0.107261000
1	-3.671545000	-4.327957000	1.387288000
1	-3.170998000	-2.996200000	-0.603994000
1	-1.695620000	-4.034109000	-2.439243000
1	-3.209839000	-4.341548000	-3.316032000
1	-6.011314000	-5.464617000	2.222488000
1	-3.031520000	-6.659357000	1.489013000
1	-3.570563000	-1.954744000	-2.952463000
6	-1.147127000	-6.323659000	-0.819857000
6	-0.410474000	-7.155565000	0.229340000
6	0.670166000	-6.325169000	0.892403000
6	1.554114000	-5.719763000	-0.185885000
6	0.707178000	-4.863032000	-1.125736000
6	1.480028000	-4.189771000	-2.262423000
8	-1.883743000	-5.338172000	-0.155997000
8	-1.342624000	-7.636427000	1.174988000
8	1.385806000	-7.185356000	1.745486000
8	-0.237504000	-5.759005000	-1.729533000
8	1.863415000	-2.877873000	-1.919876000
1	1.985218000	-6.530366000	-0.792799000
1	-1.808973000	-6.962112000	-1.415825000
1	0.077359000	-7.994650000	-0.288665000
1	0.193939000	-5.505516000	1.453944000
1	0.172834000	-4.082823000	-0.565411000
1	2.393497000	-4.750944000	-2.491327000
1	0.836135000	-4.200764000	-3.152371000
1	-0.819065000	-7.986592000	1.909558000
1	2.104916000	-6.679716000	2.163933000
1	1.052523000	-2.297892000	-1.911321000
6	3.878019000	-5.504817000	0.275515000
6	4.657172000	-5.350254000	1.580791000
6	4.901418000	-3.884236000	1.869043000
6	5.543194000	-3.229276000	0.655345000
6	4.640856000	-3.424732000	-0.562487000
6	5.145555000	-2.804123000	-1.870490000
8	2.586225000	-5.004772000	0.463550000
8	3.930736000	-5.973598000	2.618557000
8	5.723552000	-3.799947000	3.007959000
8	4.528217000	-4.839941000	-0.775586000
8	4.260081000	-1.817518000	-2.369219000
1	6.526643000	-3.684987000	0.463729000
1	3.843223000	-6.562278000	-0.009729000
1	5.633338000	-5.839463000	1.443909000
1	3.928702000	-3.395845000	2.039543000
1	3.649705000	-3.008790000	-0.342077000
1	6.115359000	-2.318871000	-1.723755000

1	5.272498000	-3.618385000	-2.595631000
1	4.332640000	-5.683706000	3.448768000
1	5.936101000	-2.862328000	3.157703000
1	3.341004000	-2.177479000	-2.325683000
6	6.944713000	-1.273118000	0.865022000
6	7.095439000	-0.268955000	2.003471000
6	6.087491000	0.862824000	1.851859000
6	6.177272000	1.427150000	0.443983000
6	6.005433000	0.310992000	-0.584673000
6	6.012668000	0.776228000	-2.043817000
8	5.692430000	-1.865038000	1.001293000
8	6.921770000	-0.955003000	3.224246000
8	6.390561000	1.821375000	2.833552000
8	7.073928000	-0.615287000	-0.377144000
8	4.697523000	0.795324000	-2.575908000
1	7.169140000	1.884702000	0.315012000
1	7.742797000	-2.023946000	0.899355000
1	8.103128000	0.167198000	1.938742000
1	5.076823000	0.444018000	1.993866000
1	5.051843000	-0.201837000	-0.411756000
1	6.414114000	1.787447000	-2.152322000
1	6.650651000	0.091904000	-2.615905000
1	6.883154000	-0.282078000	3.917269000
1	6.030265000	2.680712000	2.553233000
1	4.455450000	-0.153866000	-2.696250000
6	5.523382000	3.595669000	-0.375345000
6	5.355665000	4.807299000	0.539038000
6	3.893518000	5.108151000	0.787632000
6	3.152539000	5.215027000	-0.535347000
6	3.362120000	3.931528000	-1.334360000
6	2.722090000	3.914157000	-2.707673000
8	5.169887000	2.426005000	0.325795000
8	6.037215000	4.550608000	1.749100000
8	3.836129000	6.297617000	1.529385000
8	4.766455000	3.735595000	-1.541860000
8	2.670885000	2.606312000	-3.241308000
1	3.527676000	6.083374000	-1.098398000
1	6.566961000	3.528466000	-0.700133000
1	5.796003000	5.673978000	0.023718000
1	3.451724000	4.264145000	1.344860000
1	2.943000000	3.094943000	-0.761830000
1	1.692633000	4.275321000	-2.630093000
1	3.280529000	4.592261000	-3.368962000
1	5.809871000	5.278375000	2.345228000
1	2.918054000	6.625146000	1.522132000
1	3.546568000	2.183308000	-3.165228000
6	1.042628000	6.363153000	-0.859213000
6	0.315632000	7.197393000	0.195959000
6	-0.736897000	6.354355000	0.888823000
6	-1.627925000	5.711023000	-0.161374000
6	-0.778291000	4.858902000	-1.101487000
6	-1.555033000	4.129699000	-2.199144000
8	1.794023000	5.393398000	-0.187013000

8	1.266796000	7.692595000	1.116499000
8	-1.456650000	7.205403000	1.746994000
8	0.129156000	5.767187000	-1.743915000
8	-1.859653000	2.803724000	-1.824485000
1	-2.093586000	6.497435000	-0.774416000
1	1.691165000	6.997941000	-1.473741000
1	-0.192346000	8.027361000	-0.316633000
1	-0.232319000	5.552360000	1.451151000
1	-0.207150000	4.109999000	-0.535385000
1	-2.507334000	4.635263000	-2.396247000
1	-0.950112000	4.160469000	-3.115373000
1	0.756524000	8.055788000	1.854317000
1	-2.133996000	6.678280000	2.207433000
1	-1.027078000	2.282201000	-1.797880000
6	-3.924672000	5.472291000	0.389392000
6	-4.663862000	5.243410000	1.705012000
6	-4.901570000	3.763137000	1.931209000
6	-5.558953000	3.167709000	0.694762000
6	-4.674997000	3.429489000	-0.521916000
6	-5.169994000	2.839717000	-1.845857000
8	-2.620966000	4.975813000	0.519878000
8	-3.906777000	5.823509000	2.747012000
8	-5.708092000	3.632337000	3.075258000
8	-4.590026000	4.854480000	-0.678883000
8	-4.355799000	1.764007000	-2.271407000
1	-6.539031000	3.643180000	0.537006000
1	-3.904098000	6.543751000	0.159729000
1	-5.645580000	5.733548000	1.620952000
1	-3.926319000	3.268429000	2.067987000
1	-3.673101000	3.029267000	-0.325754000
1	-6.184642000	2.442481000	-1.748979000
1	-5.179356000	3.649450000	-2.588077000
1	-4.293880000	5.510860000	3.575917000
1	-5.970024000	2.697909000	3.158280000
1	-3.422272000	2.057011000	-2.241163000
6	-6.988049000	1.232408000	0.775853000
6	-7.231991000	0.238587000	1.907156000
6	-6.287838000	-0.941592000	1.779823000
6	-6.366430000	-1.514227000	0.374481000
6	-6.107586000	-0.428277000	-0.668486000
6	-6.210349000	-0.885920000	-2.131922000
8	-5.720133000	1.789282000	0.960959000
8	-7.060919000	0.911062000	3.138302000
8	-6.660775000	-1.890159000	2.749952000
8	-7.095197000	0.594484000	-0.469911000
8	-4.973010000	-0.840188000	-2.803433000
1	-7.373603000	-1.933429000	0.221876000
1	-7.757223000	2.014057000	0.784309000
1	-8.261613000	-0.135727000	1.808786000
1	-5.258401000	-0.583905000	1.943542000
1	-5.105901000	-0.008495000	-0.514606000
1	-6.568389000	-1.915624000	-2.208506000
1	-6.948404000	-0.236604000	-2.621728000

1	-7.052111000	0.228440000	3.822942000
1	-6.294786000	-2.752327000	2.484887000
1	-4.681765000	0.092429000	-2.780733000
8	-0.306242000	-1.387371000	-1.887821000
1	-0.129079000	-0.441299000	-2.016086000
1	-1.144196000	-1.601485000	-2.342620000
8	0.527784000	1.455401000	-1.868994000
1	1.096221000	1.257246000	-1.099709000
1	1.175429000	1.753319000	-2.538556000
8	2.782367000	0.800991000	-0.390011000
1	3.277588000	1.310902000	0.264819000
1	3.355296000	0.848434000	-1.177208000
**γ-CD–4H_2O(a)**			
6	-6.876149000	-0.031851000	-0.346009000
6	-7.432780000	-1.108269000	0.583197000
6	-6.371872000	-2.145828000	0.883569000
6	-5.781640000	-2.675876000	-0.412804000
6	-5.251089000	-1.514452000	-1.255544000
6	-4.730996000	-1.937445000	-2.614572000
8	-5.913008000	0.701463000	0.354433000
8	-7.881789000	-0.484349000	1.768772000
8	-6.983657000	-3.158054000	1.640493000
8	-6.329812000	-0.604676000	-1.502500000
8	-3.954226000	-0.927446000	-3.228935000
1	-6.561478000	-3.211485000	-0.975997000
1	-7.686284000	0.623766000	-0.685763000
1	-8.266605000	-1.606346000	0.066868000
1	-5.557496000	-1.658320000	1.444202000
1	-4.447194000	-1.003908000	-0.705859000
1	-4.093385000	-2.818445000	-2.500622000
1	-5.588113000	-2.210463000	-3.245861000
1	-8.080510000	-1.201570000	2.387554000
1	-6.358764000	-3.900676000	1.728475000
1	-4.379904000	-0.055336000	-3.105294000
6	-4.709043000	-4.838558000	-0.598717000
6	-4.464036000	-5.851343000	0.520600000
6	-3.065848000	-5.690148000	1.088933000
6	-2.063364000	-5.718151000	-0.053395000
6	-2.397447000	-4.610548000	-1.050016000
6	-1.434347000	-4.478696000	-2.228224000
8	-4.752612000	-3.564875000	-0.024366000
8	-5.450865000	-5.675238000	1.516466000
8	-2.864236000	-6.743488000	1.997221000
8	-3.697111000	-4.921999000	-1.570707000
8	-0.395531000	-3.573629000	-1.932400000
1	-2.158698000	-6.676628000	-0.586081000
1	-5.648659000	-5.060564000	-1.117565000
1	-4.535206000	-6.857976000	0.082986000
1	-2.997093000	-4.709614000	1.587420000
1	-2.436822000	-3.638032000	-0.538512000
1	-0.964598000	-5.439023000	-2.462302000
1	-2.019037000	-4.154805000	-3.101832000

1	-5.161418000	-6.200710000	2.275619000
1	-1.930083000	-6.737129000	2.274390000
1	-0.806883000	-2.710374000	-1.741238000
6	0.133833000	-6.619124000	0.192752000
6	0.940618000	-6.960450000	1.445940000
6	1.896827000	-5.836648000	1.791349000
6	2.713708000	-5.492308000	0.555761000
6	1.770235000	-5.080430000	-0.571532000
6	2.445633000	-4.650251000	-1.876845000
8	-0.778193000	-5.608950000	0.518549000
8	0.041870000	-7.231891000	2.501303000
8	2.703083000	-6.281770000	2.854507000
8	0.969436000	-6.236486000	-0.864683000
8	2.474290000	-3.245238000	-2.024266000
1	3.272758000	-6.382583000	0.229819000
1	-0.399058000	-7.512084000	-0.154533000
1	1.543878000	-7.851738000	1.216183000
1	1.311668000	-4.947182000	2.075752000
1	1.120189000	-4.262963000	-0.243050000
1	3.486454000	-4.988796000	-1.906485000
1	1.894922000	-5.123070000	-2.699224000
1	0.574121000	-7.261969000	3.308191000
1	3.382448000	-5.605496000	3.026313000
1	1.565128000	-2.926122000	-2.160904000
6	4.972636000	-4.726835000	0.757085000
6	5.730500000	-4.091248000	1.919382000
6	5.657227000	-2.575811000	1.847749000
6	6.073029000	-2.122782000	0.457668000
6	5.205068000	-2.810761000	-0.590179000
6	5.487386000	-2.398639000	-2.035911000
8	3.611658000	-4.469030000	0.935900000
8	5.189363000	-4.583675000	3.127743000
8	6.503286000	-2.072356000	2.851583000
8	5.433742000	-4.220306000	-0.469971000
8	4.541999000	-1.451301000	-2.484284000
1	7.125027000	-2.403768000	0.293862000
1	5.160411000	-5.806513000	0.731358000
1	6.787890000	-4.379549000	1.823370000
1	4.612143000	-2.265729000	2.009282000
1	4.149444000	-2.596475000	-0.387988000
1	6.473059000	-1.934987000	-2.141241000
1	5.465214000	-3.306871000	-2.652546000
1	5.563186000	-4.041912000	3.835935000
1	6.710034000	-1.143777000	2.643485000
1	3.679021000	-1.906176000	-2.458864000
6	6.957048000	0.026999000	-0.169125000
6	7.397284000	1.107133000	0.815625000
6	6.280245000	2.108392000	1.023459000
6	5.822280000	2.651800000	-0.318054000
6	5.439030000	1.509656000	-1.261773000
6	5.139106000	2.004868000	-2.665778000
8	5.935325000	-0.715091000	0.429479000
8	7.755084000	0.491132000	2.035990000

8	6.782898000	3.116359000	1.864344000
8	6.544125000	0.606839000	-1.376902000
8	4.381368000	1.084470000	-3.424292000
1	6.641084000	3.239827000	-0.761778000
1	7.804296000	-0.620828000	-0.422690000
1	8.259614000	1.634225000	0.381620000
1	5.426049000	1.586902000	1.484462000
1	4.559716000	0.983868000	-0.863952000
1	4.551534000	2.926548000	-2.606201000
1	6.096227000	2.229439000	-3.158171000
1	7.853355000	1.209320000	2.677261000
1	6.176905000	3.878081000	1.833790000
1	4.662492000	0.172957000	-3.199572000
6	4.642346000	4.741842000	-0.652159000
6	4.416365000	5.792561000	0.435716000
6	3.040238000	5.621920000	1.049120000
6	2.009687000	5.613778000	-0.065589000
6	2.309041000	4.492850000	-1.058776000
6	1.319499000	4.415682000	-2.227877000
8	4.713279000	3.489746000	-0.037837000
8	5.431124000	5.669418000	1.410298000
8	2.850143000	6.695333000	1.937987000
8	3.604923000	4.786904000	-1.601227000
8	0.403340000	3.344757000	-2.077002000
1	2.082398000	6.561707000	-0.619820000
1	5.564254000	4.954933000	-1.205712000
1	4.456838000	6.784880000	-0.037166000
1	3.001987000	4.652659000	1.571217000
1	2.331410000	3.515452000	-0.559158000
1	0.734927000	5.338713000	-2.299627000
1	1.905399000	4.298946000	-3.148570000
1	5.155444000	6.226447000	2.152231000
1	1.949124000	6.643156000	2.303136000
1	0.940676000	2.504462000	-2.219131000
6	-0.134667000	6.576374000	0.310916000
6	-0.914105000	6.855153000	1.595316000
6	-1.892236000	5.733590000	1.879384000
6	-2.738922000	5.494071000	0.639745000
6	-1.829257000	5.153682000	-0.540714000
6	-2.559194000	4.843391000	-1.851518000
8	0.732936000	5.505437000	0.533523000
8	0.005683000	7.030018000	2.651014000
8	-2.672438000	6.123352000	2.981488000
8	-1.004661000	6.305063000	-0.760192000
8	-2.281090000	3.531758000	-2.330534000
1	-3.294535000	6.412048000	0.395146000
1	0.426041000	7.471588000	0.020259000
1	-1.496352000	7.775031000	1.434552000
1	-1.325312000	4.812650000	2.091109000
1	-1.199646000	4.291202000	-0.283204000
1	-3.642106000	4.900442000	-1.714999000
1	-2.259211000	5.593724000	-2.592203000
1	-0.509988000	7.019822000	3.469027000

1	-3.365367000	5.453412000	3.120175000
1	-1.309388000	3.467471000	-2.483202000
6	-4.998963000	4.709415000	0.797124000
6	-5.753279000	4.033036000	1.937117000
6	-5.670953000	2.521388000	1.815465000
6	-6.069350000	2.104262000	0.408696000
6	-5.204274000	2.834308000	-0.616719000
6	-5.471899000	2.474912000	-2.082722000
8	-3.636508000	4.452336000	0.962739000
8	-5.211271000	4.489499000	3.158341000
8	-6.527291000	1.980576000	2.789793000
8	-5.455429000	4.234217000	-0.446680000
8	-4.426835000	1.700216000	-2.633490000
1	-7.123498000	2.376281000	0.244017000
1	-5.192974000	5.787982000	0.801020000
1	-6.811977000	4.318862000	1.850749000
1	-4.626355000	2.210366000	1.979081000
1	-4.147433000	2.619853000	-0.407138000
1	-6.383505000	1.881444000	-2.190630000
1	-5.609137000	3.411443000	-2.638165000
1	-5.591008000	3.934965000	3.853453000
1	-6.750517000	1.068531000	2.532421000
1	-3.644554000	2.272272000	-2.729248000
8	-1.640062000	-1.055865000	-1.849952000
1	-1.572632000	-0.252444000	-1.289833000
1	-2.441270000	-0.941832000	-2.413052000
8	1.955576000	1.304419000	-2.184360000
1	1.556899000	0.424672000	-2.348958000
1	2.780444000	1.295120000	-2.717456000
8	0.821850000	-1.087949000	-2.889682000
1	-0.133499000	-1.032738000	-2.645479000
1	0.851949000	-1.222453000	-3.842464000
8	-1.362428000	1.419302000	-0.630171000
1	-0.477540000	1.808834000	-0.626914000
1	-1.898552000	2.075875000	-1.102482000
**γ-CD–5H_2O(a)**			
6	6.894554000	0.277195000	-0.554090000
6	7.435839000	1.410202000	0.315667000
6	6.337829000	2.399631000	0.644341000
6	5.643009000	2.852782000	-0.628706000
6	5.138023000	1.633778000	-1.398210000
6	4.488203000	1.981218000	-2.721672000
8	6.006136000	-0.482090000	0.213693000
8	7.974232000	0.844530000	1.493768000
8	6.937542000	3.463388000	1.337572000
8	6.254984000	0.785362000	-1.694324000
8	3.776131000	0.889821000	-3.267713000
1	6.349844000	3.421928000	-1.252120000
1	7.722402000	-0.343662000	-0.916248000
1	8.215767000	1.936454000	-0.254419000
1	5.584168000	1.886930000	1.264858000
1	4.413343000	1.094296000	-0.771196000

1	3.771574000	2.793121000	-2.566699000
1	5.265571000	2.335411000	-3.414257000
1	8.169295000	1.589661000	2.079815000
1	6.282733000	4.177916000	1.441610000
1	4.341985000	0.096482000	-3.268841000
6	4.422697000	4.943369000	-0.784778000
6	4.202304000	5.940289000	0.353299000
6	2.886488000	5.633942000	1.036692000
6	1.781634000	5.614402000	-0.006297000
6	2.088919000	4.588663000	-1.093987000
6	1.059036000	4.537595000	-2.225534000
8	4.571652000	3.676872000	-0.211917000
8	5.283273000	5.856970000	1.259933000
8	2.675017000	6.628678000	2.010416000
8	3.340920000	4.971737000	-1.681517000
8	0.015059000	3.634875000	-1.949521000
1	1.731388000	6.600007000	-0.493878000
1	5.309429000	5.211677000	-1.370232000
1	4.143853000	6.948559000	-0.082358000
1	2.951091000	4.634596000	1.494014000
1	2.176331000	3.583301000	-0.657145000
1	0.605884000	5.524442000	-2.370614000
1	1.595128000	4.268121000	-3.147006000
1	4.991494000	6.319850000	2.058543000
1	1.849903000	6.424320000	2.485723000
1	0.398749000	2.734759000	-1.952090000
6	-0.335098000	6.407085000	0.716513000
6	-1.045089000	6.378078000	2.067334000
6	-1.962281000	5.172469000	2.177186000
6	-2.861234000	5.148184000	0.951362000
6	-2.002881000	5.099556000	-0.306291000
6	-2.771678000	4.985904000	-1.620833000
8	0.574255000	5.347016000	0.674126000
8	-0.067661000	6.393431000	3.086347000
8	-2.685159000	5.316903000	3.373985000
8	-1.254745000	6.321766000	-0.339583000
8	-2.870221000	3.636016000	-2.031972000
1	-3.458069000	6.071731000	0.933461000
1	0.183923000	7.364939000	0.594840000
1	-1.676555000	7.277432000	2.126408000
1	-1.350185000	4.255356000	2.179494000
1	-1.311032000	4.251994000	-0.255830000
1	-3.794471000	5.363401000	-1.524746000
1	-2.242375000	5.589130000	-2.368153000
1	-0.527273000	6.202424000	3.915034000
1	-3.418484000	4.676729000	3.376673000
1	-1.992220000	3.343272000	-2.341159000
6	-5.097700000	4.280576000	0.920483000
6	-5.864853000	3.577839000	2.038582000
6	-5.840570000	2.080220000	1.836364000
6	-6.306428000	1.737647000	0.431838000
6	-5.454712000	2.476586000	-0.600676000
6	-5.860990000	2.274685000	-2.065500000

8	-3.723841000	4.025388000	1.071067000
8	-5.297509000	3.944874000	3.278620000
8	-6.673374000	1.501862000	2.812130000
8	-5.564251000	3.881890000	-0.335204000
8	-4.780874000	1.860967000	-2.876063000
1	-7.367101000	2.014736000	0.321202000
1	-5.280127000	5.359149000	0.976429000
1	-6.911229000	3.912402000	1.978516000
1	-4.800727000	1.725607000	1.935420000
1	-4.415249000	2.144147000	-0.491933000
1	-6.625978000	1.503477000	-2.168392000
1	-6.279883000	3.223380000	-2.423705000
1	-5.697415000	3.369199000	3.944854000
1	-6.891810000	0.601177000	2.517270000
1	-4.065030000	2.518803000	-2.762434000
6	-6.996734000	-0.396122000	-0.469067000
6	-7.522167000	-1.594932000	0.316298000
6	-6.397698000	-2.565343000	0.612512000
6	-5.628085000	-2.904445000	-0.655207000
6	-5.144020000	-1.611610000	-1.309704000
6	-4.380571000	-1.775653000	-2.608248000
8	-6.158793000	0.333684000	0.371610000
8	-8.105834000	-1.116502000	1.509848000
8	-6.974084000	-3.703040000	1.202133000
8	-6.300669000	-0.819472000	-1.613236000
8	-3.661737000	-0.596375000	-2.924249000
1	-6.282372000	-3.454872000	-1.348583000
1	-7.831987000	0.221529000	-0.820738000
1	-8.266931000	-2.110947000	-0.308113000
1	-5.690264000	-2.072098000	1.299429000
1	-4.494614000	-1.077605000	-0.601941000
1	-3.653573000	-2.585385000	-2.500454000
1	-5.082743000	-2.038549000	-3.411076000
1	-8.290793000	-1.899386000	2.047120000
1	-6.290262000	-4.393184000	1.270959000
1	-4.246580000	0.196081000	-2.873280000
6	-4.288156000	-4.900955000	-0.925672000
6	-4.074083000	-6.016258000	0.100926000
6	-2.788641000	-5.777392000	0.869631000
6	-1.657516000	-5.577173000	-0.125278000
6	-1.975276000	-4.379808000	-1.017795000
6	-0.903925000	-4.026983000	-2.048164000
8	-4.558364000	-3.725651000	-0.224836000
8	-5.194384000	-6.063091000	0.959559000
8	-2.573886000	-6.900587000	1.692442000
8	-3.156023000	-4.754011000	-1.746306000
8	-0.027718000	-3.021730000	-1.574545000
1	-1.595797000	-6.455898000	-0.784486000
1	-5.117537000	-5.153341000	-1.596630000
1	-3.969340000	-6.964713000	-0.446941000
1	-2.897052000	-4.858701000	1.467208000
1	-2.182697000	-3.481808000	-0.421369000
1	-0.290957000	-4.905291000	-2.284255000

1	-1.416556000	-3.707507000	-2.965824000
1	-4.948848000	-6.649021000	1.689113000
1	-1.753067000	-6.758107000	2.195348000
1	-0.562568000	-2.217735000	-1.356220000
6	0.436623000	-6.508524000	0.444999000
6	1.212067000	-6.711212000	1.745124000
6	2.174691000	-5.563570000	1.991136000
6	3.008033000	-5.340188000	0.737546000
6	2.070956000	-5.064315000	-0.434247000
6	2.750117000	-4.704398000	-1.755726000
8	-0.458838000	-5.448054000	0.613695000
8	0.277756000	-6.860602000	2.791774000
8	2.969962000	-5.905238000	3.099124000
8	1.304873000	-6.258273000	-0.632558000
8	2.585860000	-3.325616000	-2.062028000
1	3.589428000	-6.247674000	0.515688000
1	-0.102243000	-7.428903000	0.193868000
1	1.812726000	-7.626296000	1.626909000
1	1.591961000	-4.647711000	2.181234000
1	1.393115000	-4.243391000	-0.170727000
1	3.828015000	-4.887689000	-1.710330000
1	2.318732000	-5.334412000	-2.543595000
1	0.777697000	-6.828718000	3.618395000
1	3.640206000	-5.209539000	3.220527000
1	1.620603000	-3.129468000	-2.083980000
6	5.246830000	-4.486162000	0.857868000
6	5.977287000	-3.728904000	1.962765000
6	5.847951000	-2.229700000	1.759651000
6	6.250016000	-1.869008000	0.339114000
6	5.428377000	-2.686858000	-0.652732000
6	5.701045000	-2.390261000	-2.132169000
8	3.876285000	-4.256979000	1.006390000
8	5.442650000	-4.135430000	3.204973000
8	6.669648000	-1.604758000	2.714164000
8	5.701919000	-4.070414000	-0.408290000
8	4.633150000	-1.678064000	-2.732772000
1	7.321437000	-2.084379000	0.202911000
1	5.467948000	-5.557651000	0.922526000
1	7.044333000	-3.988043000	1.894780000
1	4.790639000	-1.945331000	1.888909000
1	4.368541000	-2.487132000	-0.463215000
1	6.594590000	-1.774998000	-2.265822000
1	5.869099000	-3.346678000	-2.642769000
1	5.800775000	-3.532814000	3.870915000
1	6.864750000	-0.703748000	2.402609000
1	3.862492000	-2.280963000	-2.757506000
8	1.182704000	1.148389000	-2.242338000
1	1.454743000	0.547359000	-1.520703000
1	1.934342000	1.078405000	-2.858678000
8	-1.779909000	-1.109518000	-0.943474000
1	-1.741093000	-0.379603000	-0.301533000
1	-2.359719000	-0.763965000	-1.648157000
8	-1.555854000	1.339007000	-2.795874000

1	-0.629704000	1.040007000	-2.741259000
1	-2.065672000	0.630831000	-3.212474000
8	2.612289000	-0.691633000	-0.763398000
1	2.337598000	-1.615722000	-0.690350000
1	3.338839000	-0.721473000	-1.406719000
1	-2.065713000	1.695372000	-0.981852000
8	-2.131891000	1.491632000	-0.031993000
1	-2.560846000	2.258049000	0.371269000
**γ-CD–6H_2O(a)**			
6	-6.357009000	1.792475000	-0.342213000
6	-7.172638000	0.892500000	0.585011000
6	-6.491625000	-0.443857000	0.798050000
6	-6.132822000	-1.068205000	-0.540540000
6	-5.278910000	-0.084544000	-1.333362000
6	-4.875134000	-0.543832000	-2.717874000
8	-5.193232000	2.223841000	0.318109000
8	-7.357241000	1.566726000	1.813048000
8	-7.376718000	-1.239789000	1.542023000
8	-6.030139000	1.119373000	-1.522051000
8	-3.919875000	0.333268000	-3.276554000
1	-7.052635000	-1.303217000	-1.097592000
1	-6.962895000	2.654474000	-0.641424000
1	-8.141524000	0.704458000	0.098754000
1	-5.547805000	-0.271700000	1.342834000
1	-4.362469000	0.119129000	-0.759898000
1	-4.421984000	-1.537866000	-2.656789000
1	-5.773968000	-0.607144000	-3.348366000
1	-7.778448000	0.929330000	2.407285000
1	-7.063232000	-2.162464000	1.517111000
1	-4.197542000	1.254758000	-3.113012000
6	-5.742819000	-3.436756000	-0.897258000
6	-5.911471000	-4.518619000	0.168333000
6	-4.581086000	-4.755412000	0.850477000
6	-3.529340000	-5.059804000	-0.200855000
6	-3.438951000	-3.931057000	-1.226191000
6	-2.438742000	-4.189243000	-2.361132000
8	-5.418671000	-2.247734000	-0.233115000
8	-6.894531000	-4.100641000	1.094536000
8	-4.749419000	-5.823369000	1.751454000
8	-4.741848000	-3.794314000	-1.814543000
8	-1.208053000	-3.539045000	-2.142262000
1	-3.813219000	-5.978224000	-0.737532000
1	-6.663351000	-3.317295000	-1.480501000
1	-6.222670000	-5.448438000	-0.330064000
1	-4.288191000	-3.832641000	1.375076000
1	-3.159415000	-2.991331000	-0.729668000
1	-2.227259000	-5.260967000	-2.444979000
1	-2.909635000	-3.856178000	-3.296486000
1	-6.819808000	-4.704081000	1.847686000
1	-3.903387000	-5.975850000	2.209292000
1	-1.369735000	-2.574483000	-2.220293000
6	-1.750549000	-6.527898000	0.416204000

6	-1.103706000	-6.828775000	1.765710000
6	0.088372000	-5.916761000	1.997295000
6	1.005460000	-5.999952000	0.788178000
6	0.233144000	-5.629808000	-0.472489000
6	1.059380000	-5.614700000	-1.756131000
8	-2.312975000	-5.251807000	0.487914000
8	-2.081254000	-6.681181000	2.774537000
8	0.715546000	-6.346713000	3.179206000
8	-0.806882000	-6.604036000	-0.620476000
8	1.544518000	-4.312943000	-2.016459000
1	1.370394000	-7.032218000	0.684091000
1	-2.517479000	-7.278296000	0.190906000
1	-0.733657000	-7.864540000	1.735962000
1	-0.272964000	-4.878614000	2.084025000
1	-0.214227000	-4.635388000	-0.361961000
1	1.932686000	-6.269840000	-1.678802000
1	0.419069000	-5.971604000	-2.571896000
1	-1.608536000	-6.685772000	3.618043000
1	1.546918000	-5.851778000	3.288975000
1	0.789580000	-3.756429000	-2.285228000
6	3.383620000	-5.694301000	0.966085000
6	4.216749000	-5.172295000	2.134417000
6	4.573494000	-3.714475000	1.937733000
6	5.208224000	-3.529248000	0.570884000
6	4.256855000	-4.039609000	-0.510679000
6	4.764652000	-3.934223000	-1.955093000
8	2.099454000	-5.125348000	1.024908000
8	3.497233000	-5.377121000	3.332466000
8	5.454602000	-3.353582000	2.974277000
8	4.007081000	-5.430335000	-0.256409000
8	3.912431000	-3.162449000	-2.774981000
1	6.159446000	-4.083768000	0.533699000
1	3.303603000	-6.784671000	1.033802000
1	5.155007000	-5.747033000	2.146344000
1	3.652272000	-3.108527000	1.960383000
1	3.324941000	-3.469027000	-0.441044000
1	5.745327000	-3.457053000	-2.005979000
1	4.862931000	-4.956264000	-2.342929000
1	3.971154000	-4.893158000	4.022598000
1	5.943246000	-2.562200000	2.688186000
1	3.020557000	-3.558942000	-2.711306000
6	6.534154000	-1.702900000	-0.274358000
6	7.369873000	-0.746848000	0.574374000
6	6.648650000	0.569086000	0.790545000
6	6.147376000	1.125824000	-0.534925000
6	5.265224000	0.071262000	-1.199662000
6	4.647614000	0.450122000	-2.529176000
8	5.447746000	-2.139103000	0.484384000
8	7.648855000	-1.384066000	1.804130000
8	7.554349000	1.434227000	1.426621000
8	6.092379000	-1.074759000	-1.447725000
8	3.622686000	-0.464123000	-2.874854000
1	7.003200000	1.358199000	-1.186891000

1	7.151099000	-2.552444000	-0.590294000
1	8.298838000	-0.532951000	0.024636000
1	5.763170000	0.380490000	1.420280000
1	4.447195000	-0.195373000	-0.515968000
1	4.197223000	1.443394000	-2.449388000
1	5.429319000	0.480457000	-3.300763000
1	8.076364000	-0.717170000	2.359101000
1	7.176737000	2.332156000	1.425640000
1	3.951180000	-1.388228000	-2.796514000
6	5.705666000	3.471523000	-0.927582000
6	5.857585000	4.616579000	0.073807000
6	4.528165000	4.893425000	0.748376000
6	3.463590000	5.106386000	-0.314084000
6	3.386441000	3.878850000	-1.220094000
6	2.347897000	3.958106000	-2.343439000
8	5.441765000	2.306518000	-0.204367000
8	6.850352000	4.270038000	1.017110000
8	4.698989000	6.030820000	1.559726000
8	4.675475000	3.751955000	-1.841194000
8	1.173180000	3.247352000	-2.010790000
1	3.742418000	5.970681000	-0.935760000
1	6.618333000	3.359878000	-1.524586000
1	6.153848000	5.517216000	-0.484212000
1	4.247291000	4.012284000	1.346351000
1	3.177620000	2.980495000	-0.624470000
1	2.056140000	4.996605000	-2.535211000
1	2.814038000	3.555034000	-3.253083000
1	6.778804000	4.919374000	1.730984000
1	3.842514000	6.246911000	1.968763000
1	1.429482000	2.319109000	-1.746604000
6	1.652143000	6.604587000	0.115475000
6	1.033697000	7.114500000	1.419363000
6	-0.148097000	6.250692000	1.812634000
6	-1.102389000	6.151627000	0.632991000
6	-0.357253000	5.566495000	-0.563315000
6	-1.211975000	5.337308000	-1.811033000
8	2.251914000	5.370176000	0.368050000
8	2.034972000	7.123700000	2.414036000
8	-0.759987000	6.836847000	2.936665000
8	0.680671000	6.497615000	-0.895258000
8	-1.359274000	3.952755000	-2.103739000
1	-1.470132000	7.154021000	0.365711000
1	2.391308000	7.325504000	-0.251937000
1	0.662323000	8.133998000	1.234092000
1	0.218207000	5.237253000	2.040383000
1	0.092528000	4.606333000	-0.282923000
1	-2.217136000	5.748092000	-1.673994000
1	-0.735185000	5.858494000	-2.649942000
1	1.583556000	7.264637000	3.257299000
1	-1.520275000	6.284155000	3.189559000
1	-0.445809000	3.609058000	-2.265180000
6	-3.448183000	5.900922000	1.060784000
6	-4.219988000	5.261949000	2.209374000

6	-4.459929000	3.781867000	1.943944000
6	-5.075234000	3.618919000	0.562918000
6	-4.180573000	4.295655000	-0.472742000
6	-4.603493000	4.103684000	-1.930452000
8	-2.174813000	5.328741000	1.044410000
8	-3.485808000	5.469131000	3.396984000
8	-5.299224000	3.313575000	2.970394000
8	-4.122576000	5.688166000	-0.160930000
8	-3.918383000	3.005652000	-2.503223000
1	-6.070209000	4.088882000	0.556999000
1	-3.387054000	6.987067000	1.193188000
1	-5.202616000	5.753473000	2.267614000
1	-3.491966000	3.254886000	1.940162000
1	-3.174093000	3.878152000	-0.374943000
1	-5.673627000	3.896673000	-2.025627000
1	-4.394632000	5.033887000	-2.474710000
1	-3.902732000	4.926468000	4.079713000
1	-5.789428000	2.540776000	2.641079000
1	-2.983432000	3.271222000	-2.601540000
8	-1.618904000	-0.848301000	-2.300788000
1	-1.394542000	-0.450500000	-1.436060000
1	-2.319013000	-0.303707000	-2.711265000
8	1.959060000	0.932138000	-1.093334000
1	1.210564000	0.511310000	-0.621315000
1	2.344578000	0.242085000	-1.659455000
8	1.039234000	-1.633669000	-2.488027000
1	0.201013000	-1.160278000	-2.643922000
1	1.728433000	-1.195260000	-3.006128000
8	-2.257493000	1.629858000	-0.389854000
1	-1.782066000	2.341749000	-0.845673000
1	-3.181440000	1.775697000	-0.639826000
1	1.326972000	-2.245788000	-0.715085000
8	1.306634000	-2.315614000	0.257888000
1	1.465164000	-3.247412000	0.461718000
8	-0.289751000	-0.216076000	-0.015493000
1	-0.904505000	0.294659000	0.526657000
1	0.036079000	-1.014989000	0.442720000
**γ-CD–7H_2O(a)**			
6	-6.314334000	-2.286770000	-0.510001000
6	-6.443708000	-3.473397000	0.444796000
6	-5.087869000	-4.059779000	0.782821000
6	-4.329074000	-4.368633000	-0.498139000
6	-4.223246000	-3.087438000	-1.317201000
6	-3.510168000	-3.233331000	-2.642635000
8	-5.735059000	-1.206538000	0.169661000
8	-7.111654000	-3.040655000	1.613472000
8	-5.328162000	-5.203599000	1.561135000
8	-5.546677000	-2.630910000	-1.627783000
8	-3.264591000	-1.966406000	-3.222222000
1	-4.867551000	-5.137700000	-1.073449000
1	-7.304153000	-2.011294000	-0.891970000
1	-7.029954000	-4.252576000	-0.065214000

1	-4.499590000	-3.308261000	1.335975000
1	-3.692015000	-2.337416000	-0.714686000
1	-2.541101000	-3.714536000	-2.483048000
1	-4.111953000	-3.867525000	-3.309036000
1	-7.044384000	-3.775969000	2.239833000
1	-4.489327000	-5.688625000	1.670225000
1	-4.087927000	-1.446276000	-3.214441000
6	-2.599422000	-6.055913000	-0.616291000
6	-2.066275000	-6.877602000	0.558447000
6	-0.838500000	-6.203109000	1.134268000
6	0.163652000	-5.990456000	0.014661000
6	-0.453211000	-5.137892000	-1.091525000
6	0.474901000	-4.898399000	-2.287894000
8	-3.057187000	-4.838950000	-0.105125000
8	-3.080693000	-7.012531000	1.533123000
8	-0.336717000	-7.048315000	2.142611000
8	-1.595746000	-5.860635000	-1.579559000
8	1.185099000	-3.685710000	-2.177117000
1	0.422092000	-6.967119000	-0.423104000
1	-3.408035000	-6.592268000	-1.126511000
1	-1.770874000	-7.866097000	0.176565000
1	-1.127950000	-5.219033000	1.536077000
1	-0.777067000	-4.167502000	-0.687023000
1	1.216921000	-5.700832000	-2.364009000
1	-0.147193000	-4.919172000	-3.193578000
1	-2.635471000	-7.325348000	2.333643000
1	0.485990000	-6.661282000	2.490639000
1	0.518754000	-2.971601000	-2.228912000
6	2.498928000	-6.128900000	0.490191000
6	3.277056000	-5.954637000	1.792864000
6	3.779264000	-4.528347000	1.939157000
6	4.525313000	-4.150863000	0.670344000
6	3.597705000	-4.314624000	-0.529391000
6	4.193576000	-3.883739000	-1.865736000
8	1.312601000	-5.402052000	0.591763000
8	2.444301000	-6.326160000	2.870182000
8	4.596678000	-4.499446000	3.081397000
8	3.266389000	-5.703123000	-0.608266000
8	3.894330000	-2.524122000	-2.117254000
1	5.383503000	-4.826647000	0.545354000
1	2.288716000	-7.191080000	0.320379000
1	4.158921000	-6.611025000	1.743616000
1	2.911385000	-3.855255000	2.043065000
1	2.679004000	-3.733913000	-0.383746000
1	5.284776000	-3.974031000	-1.867473000
1	3.776351000	-4.533124000	-2.643190000
1	2.896515000	-6.043746000	3.676784000
1	5.087762000	-3.658939000	3.097710000
1	2.949302000	-2.455631000	-2.350945000
6	6.363233000	-2.586815000	0.612106000
6	6.914084000	-1.748684000	1.764058000
6	6.368985000	-0.341952000	1.690773000
6	6.639978000	0.255179000	0.321291000

6	6.053141000	-0.648293000	-0.765250000
6	6.328717000	-0.236444000	-2.215372000
8	4.988919000	-2.813800000	0.822777000
8	6.576415000	-2.367838000	2.987620000
8	6.980918000	0.410407000	2.710895000
8	6.606993000	-1.966060000	-0.613768000
8	5.157092000	-0.254290000	-3.006160000
1	7.726046000	0.378647000	0.181784000
1	6.887532000	-3.547457000	0.570780000
1	8.006604000	-1.703024000	1.643826000
1	5.274276000	-0.378981000	1.820890000
1	4.967240000	-0.670965000	-0.627836000
1	6.720741000	0.778914000	-2.281122000
1	7.084913000	-0.923594000	-2.615503000
1	6.787128000	-1.730289000	3.683796000
1	6.852864000	1.349568000	2.494863000
1	4.736878000	-1.129950000	-2.883819000
6	6.456935000	2.539208000	-0.438803000
6	6.561972000	3.825410000	0.380099000
6	5.188932000	4.363644000	0.737563000
6	4.315849000	4.439087000	-0.506139000
6	4.245856000	3.049641000	-1.133876000
6	3.384038000	2.918202000	-2.371953000
8	6.011574000	1.520531000	0.399867000
8	7.322706000	3.549596000	1.535892000
8	5.383837000	5.622119000	1.330119000
8	5.581916000	2.704324000	-1.524395000
8	3.128435000	1.555967000	-2.654565000
1	4.764106000	5.138248000	-1.227756000
1	7.436326000	2.288916000	-0.864965000
1	7.059687000	4.579689000	-0.248498000
1	4.709565000	3.659243000	1.437935000
1	3.873586000	2.329773000	-0.391144000
1	2.421989000	3.404002000	-2.191805000
1	3.876781000	3.418532000	-3.216625000
1	7.270063000	4.344645000	2.084159000
1	4.515432000	6.046963000	1.449309000
1	3.971419000	1.056643000	-2.751242000
6	2.514808000	6.016111000	-0.750634000
6	1.941739000	6.983149000	0.287230000
6	0.705945000	6.395690000	0.941107000
6	-0.258449000	5.958355000	-0.148866000
6	0.425448000	4.930162000	-1.046311000
6	-0.443644000	4.368334000	-2.171698000
8	3.056102000	4.919454000	-0.074853000
8	2.944716000	7.279175000	1.235919000
8	0.156144000	7.395503000	1.764953000
8	1.523922000	5.618580000	-1.663585000
8	-1.059358000	3.150239000	-1.801192000
1	-0.506260000	6.825551000	-0.779102000
1	3.285622000	6.519319000	-1.345572000
1	1.635513000	7.896313000	-0.245023000
1	0.999155000	5.508839000	1.525371000

1	0.807245000	4.087799000	-0.453710000
1	-1.246517000	5.068898000	-2.428243000
1	0.197737000	4.239219000	-3.054079000
1	2.503858000	7.750075000	1.956815000
1	-0.660846000	7.050856000	2.166936000
1	-0.355261000	2.511069000	-1.526674000
6	-2.590427000	6.211556000	0.237817000
6	-3.438649000	6.230578000	1.509293000
6	-4.006163000	4.854414000	1.795116000
6	-4.704604000	4.335269000	0.547950000
6	-3.710920000	4.314538000	-0.609681000
6	-4.256250000	3.743211000	-1.920678000
8	-1.427432000	5.475999000	0.484748000
8	-2.637396000	6.702622000	2.571225000
8	-4.886518000	4.970258000	2.885299000
8	-3.309832000	5.670742000	-0.841870000
8	-3.611963000	2.525879000	-2.275879000
1	-5.544757000	4.997273000	0.288590000
1	-2.340608000	7.238866000	-0.050161000
1	-4.283531000	6.912743000	1.329410000
1	-3.172034000	4.171460000	2.024070000
1	-2.831903000	3.724383000	-0.323207000
1	-5.323761000	3.516619000	-1.833993000
1	-4.120817000	4.498427000	-2.704336000
1	-3.134422000	6.540744000	3.384532000
1	-5.287909000	4.097496000	3.046134000
1	-2.644896000	2.704333000	-2.331180000
6	-6.544729000	2.791175000	0.742964000
6	-6.931671000	1.850188000	1.876928000
6	-6.245914000	0.504474000	1.704514000
6	-6.504994000	-0.025154000	0.302699000
6	-6.094570000	1.021615000	-0.730782000
6	-6.265978000	0.591280000	-2.191622000
8	-5.174064000	3.039582000	0.857370000
8	-6.574933000	2.459155000	3.101158000
8	-6.752318000	-0.344326000	2.703948000
8	-6.861079000	2.207522000	-0.500362000
8	-5.030945000	0.205522000	-2.772407000
1	-7.577850000	-0.251525000	0.199711000
1	-7.115868000	3.724901000	0.799032000
1	-8.018381000	1.688441000	1.825675000
1	-5.157618000	0.644950000	1.807862000
1	-5.037144000	1.249255000	-0.574431000
1	-6.934717000	-0.269012000	-2.285947000
1	-6.713836000	1.427955000	-2.742476000
1	-6.648039000	1.773451000	3.779325000
1	-6.625716000	-1.266508000	2.418524000
1	-4.492402000	1.015210000	-2.867802000
8	-0.754804000	-1.745449000	-2.013901000
1	-1.029784000	-1.762397000	-1.060210000
1	-1.569564000	-1.576576000	-2.522556000
8	0.960391000	1.713930000	-0.808955000
1	0.741898000	1.082006000	-0.096878000

1	1.704117000	1.332935000	-1.303145000
8	1.745087000	-0.819745000	-2.310065000
1	0.768168000	-0.917912000	-2.266846000
1	1.960216000	-0.017650000	-2.807657000
8	-2.974433000	0.101077000	-0.731237000
1	-2.935875000	1.046766000	-0.928610000
1	-3.672373000	-0.229444000	-1.317875000
1	2.580512000	-0.939718000	-0.756638000
8	2.851222000	-0.888473000	0.186959000
1	3.481242000	-1.609952000	0.335197000
8	0.609111000	-0.175416000	1.264704000
1	0.754839000	0.251952000	2.114759000
1	1.483793000	-0.569125000	0.996070000
8	-1.525332000	-1.813510000	0.555960000
1	-0.769410000	-1.378727000	0.992058000
1	-2.148188000	-1.082060000	0.390346000
**$7H_2O$ (cluster)**			
8	-0.451772000	-1.835357000	-2.064106000
1	-0.494223000	-2.030703000	-1.099714000
1	-1.270847000	-1.321550000	-2.176872000
8	-0.236652000	1.442135000	-0.746576000
1	0.105151000	1.196067000	0.132653000
1	0.443517000	1.120665000	-1.364713000
8	1.502543000	-0.192547000	-2.185795000
1	0.735130000	-0.853008000	-2.240660000
1	1.920778000	-0.156597000	-3.051105000
8	-2.341762000	-0.079228000	-1.155697000
1	-1.599378000	0.571307000	-1.012559000
1	-3.141251000	0.434446000	-1.298789000
1	2.314980000	-0.880454000	-0.809330000
8	2.490920000	-1.258373000	0.081828000
1	2.139762000	-2.155258000	0.052786000
8	0.783558000	0.153099000	1.491118000
1	1.089872000	0.433610000	2.358052000
1	1.525819000	-0.346674000	1.056730000
8	-1.011229000	-1.799249000	0.590698000
1	-0.475290000	-1.156541000	1.086949000
1	-1.768793000	-1.283279000	0.266583000
